# Structural Transition from Helices to Hemihelices

**DOI:** 10.1371/journal.pone.0093183

**Published:** 2014-04-23

**Authors:** Jia Liu, Jiangshui Huang, Tianxiang Su, Katia Bertoldi, David R. Clarke

**Affiliations:** 1 School of Engineering and Applied Sciences, Harvard University, Cambridge, Massachusetts, United States of America; 2 Kavli Institute, Harvard University, Cambridge, Massachusetts, United States of America; Massachusetts Institute of Technology, United States of America

## Abstract

Helices are amongst the most common structures in nature and in some cases, such as tethered plant tendrils, a more complex but related shape, the hemihelix forms. In its simplest form it consists of two helices of opposite chirality joined by a perversion. A recent, simple experiment using elastomer strips reveals that hemihelices with multiple reversals of chirality can also occur, a richness not anticipated by existing analyses. Here, we show through analysis and experiments that the transition from a helical to a hemihelical shape, as well as the number of perversions, depends on the height to width ratio of the strip's cross-section. Our findings provides the basis for the deterministic manufacture of a variety of complex three-dimensional shapes from flat strips.

## Introduction

Nature abounds with complex, three-dimensional shapes [1], [2]. Of these, the helix and spiral are amongst the most ubiquitous, often emerging during growth from initially straight or flat 2-D configurations. For instance, initially straight roots form helical shapes while attempting to penetrate more compact soils [3]. Similarly, as seed pods open, a chirality-creating mechanism turns an initially flat pod valve into a helix [4]–[6]. In other instances, the chirality can switch during growth as noted by Asa Gray [7] and Darwin [8] in their studies of plant tendrils. They noted that as a growing plant tendril circumnutates it can attach to another object and then, being fixed at both ends, its chirality reverses in between to maintain its topology as it continues to grow [9], [10]. This reversal of chirality - often referred to as a perversion - forms what we term here a simple hemihelix. More generally, we introduce the term hemihelix to describe multiple reversals in chirality connected by perversions. As pointed out by McMillen and Goriely [9], perversions have been observed in several physical systems with a literature that dates back to Ampère's letter to the French Academy of Sciences. Subsequently, the word perversion was used by J.B. Listing to describe the inversion of chirality in seashells [11] and by Maxwell in the context of light propagating in magnetic materials [12], but only recently have Goriely and Tabor rigorously defined perversions [13]. Although perversions can also be introduced manually, for instance, by the simple operation of holding one end of a helical telephone cord fixed and twisting the other in a direction counter to its initial chirality, perversions occur in nature during growth and as illustrated by the example of the attached plant tendril a single perversion forms. Interestingly, wool fibres can also form hemihelices with distributions of perversions separating alternating helical sections of opposite chiralities [14]. Recently, similar three-dimensional shapes with multiple perversions have been created by joining two strips of elastomers of different lengths [15]. Moreover, rippled patterns with periodic distributions of perversions have also been discerned along the peripheries of thin sheets, such as the edge of the gut [16] and the edges of flowers and leaves [17]–[21]. These observations raise two fundamental questions: (i) what controls whether a helix or a hemihelix forms ? and (ii) what determines the number of perversions that will form ?

**Figure 1:**
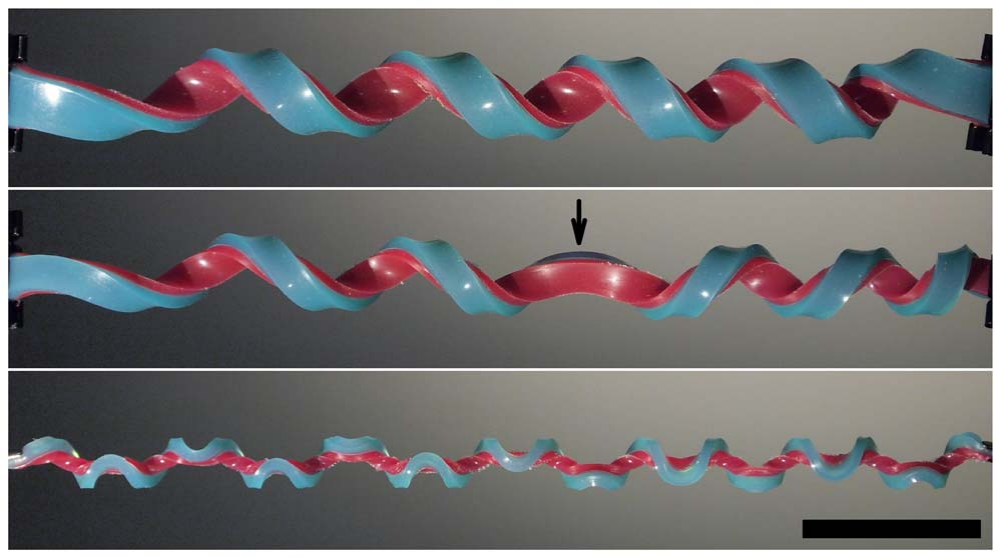
Illustration of a helix (top), a hemihelix with one perversion marked by an arrow (middle) and a hemihelix with multiple perversions (bottom). The scale bar is 5 cm, and is the same for each image. These different shapes were all produced in the same way as shown in figure 2 with the same value of pre-strain 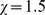 but with decreasing values of the height-to-width ratio of the bi-strip's cross-section. 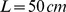, 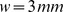, 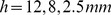).

In this work we address these two questions using a combination of experiments, numerical simulations and analyses. These show that the formation of both helices and hemihelices with periodic distributions of perversions can be fully understood in terms of competing buckling instabilities that depend on the aspect ratio of the cross-section of the bi-strip. Experiments indicate that there is a well-defined phase transition between the helix and the hemihelix and this is confirmed by an analysis based on Kirchhoff's rod theory. Our analysis also shows how the number of perversions depends on the cross-sectional aspect ratio, confirming the experimental findings discussed below and shown in Fig. 1 and those represented later on a phase diagram.

## Experimental Observations

**Figure 2:**
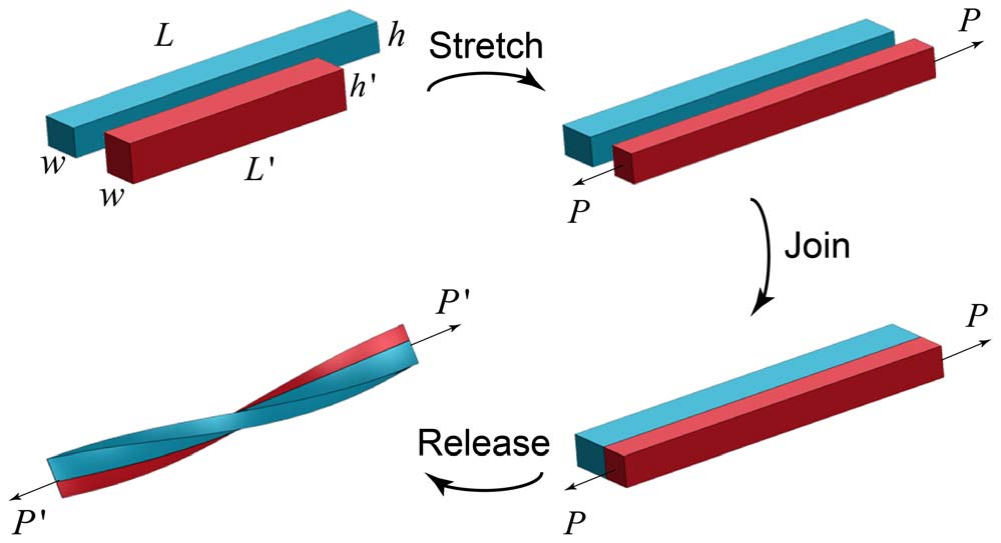
Sequence of operations leading to the spontaneous creation of hemihelices and helices. Starting with two long elastomer strips of different lengths, the shorter one is stretched to be the same length as the other. While the stretching force, P, is maintained, the two strips are joined side-by-side. Then, as the force is slowly released, the bi-strip twists and bends to create either a helix or a hemihelix.

Our observations come from a series of experiments in which two long strips of elastomer are stretched, joined and then released. The sequence of operations is shown in Fig. 2. We start with two strips of the same material (dyed to distinguish them) of the same initial width 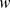 but unequal length. The short, red strip, with length 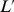 and height 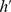, is stretched uniaxially to be equal in length to the longer, blue strip, length 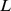 and height 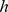. The initial heights are chosen so that after stretching the bi-strip system has a rectangular cross-section. The two strips are then glued together side-by-side along their length. At this stage, the bi-strips are flat and the red strip is under a uniaxial pre-strain, defined as 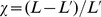. Being elastomers, volume conservation requires that the heights are related by 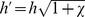. Then, in the final operation, the force stretching the ends of the bi-strip is gradually released, with the ends free to rotate. More details of the manufacturing and experimental procedures are given in *Materials and Methods*.

Upon release, the initially flat bistrips start to bend and twist out of plane and evolve towards either a helical or hemihelical shape, depending on the cross-sectional aspect ratio. As indicated by the images in Fig. 1, when the aspect ratio 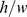 is small, we observe the formation of periodic perversions, separating helical segments of alternating chiralities, whereas when the bi-strips have a large aspect ratio, they spontaneously twist along their length to form a regular helix. Significantly, these three-dimensional shapes form spontaneously and do so irrespective of whether the release is abrupt or the ends are slowly brought together. Furthermore, it is also observed that after release, the bi-strip can be stretched straight again and released many times and each time the same shape, complete with the same number of perversions, reforms. Experiments were also performed under water to minimize gravitational effects and dampen vibrations. Video recordings, reproduced in File S1, capture the evolution of the 3D shapes, several transient features including how perversions move along the bi-strip to form a regular arrangement as well as how an initial twisting motion is reversed.

**Figure 3:**
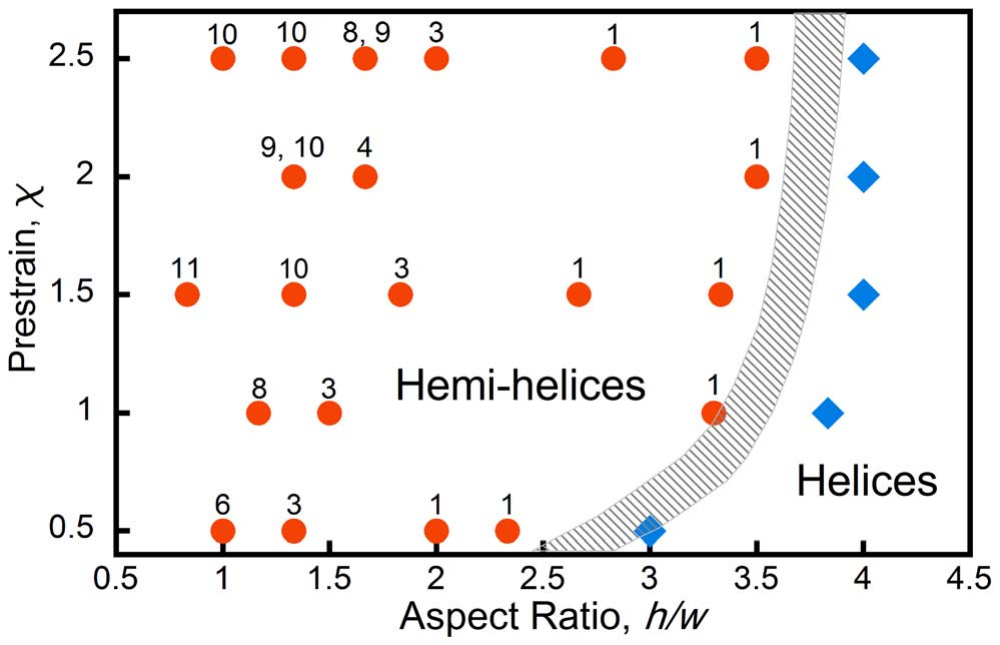
The number of perversions observed as a function of both the prestrain and the cross-section aspect ratio, 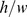. The data indicates that there is a transition between the formation of helixes at larger aspect ratios and hemihelices at smaller aspect ratios. The precise phase boundary cannot be determined with any precision experimentally and so is shown shaded but there is evidently only a weak dependence on the value of the pre-strain. In some cases, bistrips made the same way produce either one or the other of the two perversion numbers indicated.

The experimental observations indicate that the number of perversions 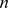 is the critical geometric parameter that distinguishes which shape forms upon release. Assuming that the perversions are uniformly distributed along the length of the bistrip, the number that form can be expected to depend on the prestrain ratio, the cross-sectional aspect ratio and the length of the bi-strip. Dimensional arguments then suggest that the number is given by: 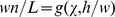. To establish how the number of perversions depends on these variables, a series of experiments were performed with different values of pre-strain and cross-sectional aspect ratio. The results of these experiments are shown in the structural phase diagram in Fig. 3 where the numbers associated with the symbols indicate the number of perversions observed. The boundary between the formation of helices and hemihelices is shown shaded. The data in Fig. 3 indicates that increasing the 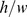 ratio drives the strip from the hemihelical configurations to helices. On the other hand, the prestrain ratio 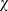 has only a weak influence on both the helix-to-hemihelix transition and the number of perversions. This phase diagram (Fig. 3) was established under experimental conditions that allowed both ends to freely rotate as the stretching force was reduced. A similar phase diagram (Fig. S5 in File S1) but notable by the absence of any helices was obtained upon unloading when the ends were constrained from rotating (see File S1 for details).

## Finite element simulations

**Figure 4:**
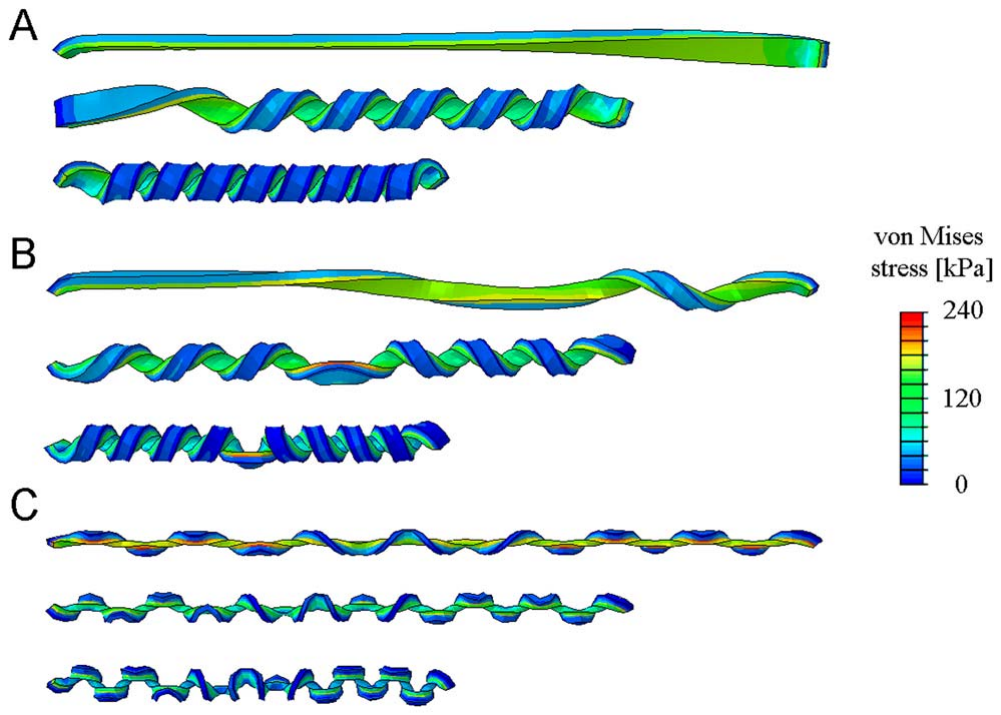
Snapshots recorded from the finite element simulations, illustrating the formation of (A) a helix, (B) a hemihelix with single perversion and (C) a hemihelix with 12 perversions. The colors represent the local values of the computed von Mises stress. The prestrain 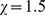 was the same in all three cases. 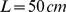, 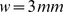, (A) 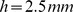, (B) 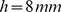, (C) 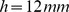. The images are taken when the end to end distances are 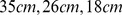. Gravity was included in the simulations and acts from left to right in these images.

Numerical simulations to explore the morphological changes occurring during the release in the bi-strip system were conducted using detailed dynamic finite element simulations. In our analysis, the non-linear response of the elastomer was captured using a hyperelastic Gent model [22]. 3D models of the bi-strip system were built and the prestretch effect was modeled by decomposition of the deformation gradient, 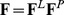, where 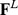 is the loading induced gradient while 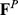 is the prestretch induced gradient, 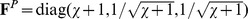 [15]. More details of the FE simulations are given in *Materials and Methods* and File S1. These fully reproduced the experimental observations. For instance, snapshots recorded at three successive stages in the release of bi-strips having three different cross-sectional aspect ratios are shown in Fig. 4. Clearly, the simulations correctly capture the principal features observed in the experiments including the formation of perversions as well as the detailed evolution of the hemihelix and the helix as unloading occurs.

## Analytical Model

**Figure 5:**
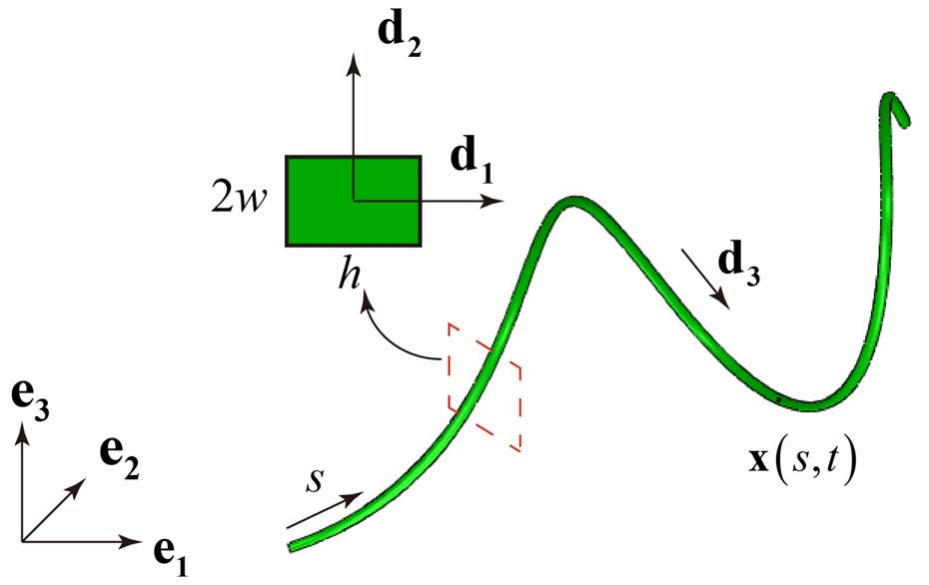
Coordinate system used in the Kirchhoff analysis together with the dimensions 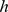 and 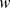 of the cross-section.

To understand the origin of the experimental and numerical results reported above, we analyze the deformation of the bi-strip system modeled as a homogeneous rod with a rectangular cross section 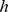 by 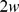 (see Fig. 5) and study its behavior using Kirchhoff rod theory [23]–[27]. Due to the pre-stretch, the equivalent homogeneous rod has a natural curvature 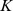 and a undeformed contour length 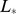 when no external forces and moments are applied (see Fig. S8 in File S1). Both 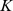 and 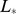 can be related to 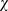, 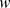 and 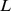 from the bi-strip system (see File S1 for details)
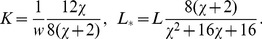
(1)The rod is then represented by a space curve 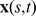, whose position depends on the arc length 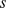 and time 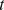 (see Fig. 5). In addition, to characterize the deformation of the rod an orthonormal local director basis 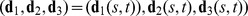 is introduced, where 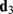 is identified as the tangent vector and 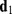 and 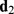 lie along the principal directions of the cross-section (Fig. 5). The condition of orthonormality implies the existence of a twist vector 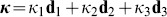 satisfying
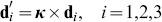
(2)where 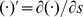, 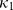 and 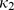 are the material curvature and 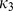 is the twist density.

Balance of force and angular momentum gives
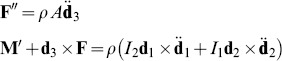
(3)where 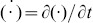, 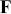 and 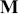 are the resultant force and moment acting on the cross section, 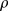 is the mass per unit volume of the rod, 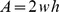 is the cross-sectional area and 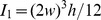 and 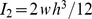 are the principal moments of inertial of the cross section.

Finally, to facilitate the analysis, the material is taken to be linear elastic, so that
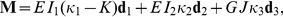
(4)where 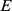 is the Young's modulus, 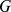 is the shear modulus, 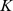 describes the local non-vanishing intrinsic curvature and is given by Eqn. (1). Moreover, 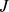 is the torsion constant, which for a rectangular cross-section can be approximated as 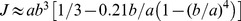 [28], where *a* = max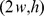 and *b* = min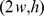.

### Stability Analysis

Our starting point is a fully stretched rod under applied tension 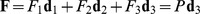. Since our experimental observations clearly show that at a critical point during the release the straight configuration becomes unstable and the rod evolves into complex 3D shapes (see videos in SI), we investigate the stability of the system during unloading. We first analyze the transition from straight to helical configurations and then the formation of hemihelices with periodic distributions of perversions. Finally, we will show that at the onset of bifurcation a helix can be described as a hemihelix with a vanishing number of perversions.

*Transition from straight to helical configurations.* A helical configuration with curvature 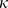 and torsion 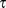 is defined by the position vector
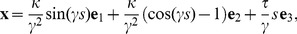
(5)where 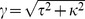 and 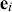 denote the global coordinate frame. To evaluate the evolution of 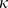 and 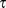 as a function of the applied force P, we minimize the total energy density (energy per unit length) of the helix
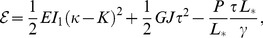
(6)where the first, second and third term are the bending energy, twisting energy and force potential, respectively. The energy minimization criterion requires that 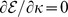 and 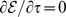, which can be solved to obtain 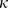 and 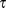 as a function of the applied force 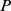. It is easy to show (see File S1 for details) that they admit real, positive solutions 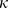 and 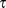 only if
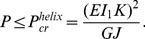
(7)Thus, our analysis predicts the formation of helical configurations during release when 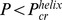.

*Transition from straight to hemihelical configurations.* For the case of small aspect ratios 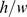, the formation of hemihelices with multiple reversal of chirality is observed in the experiments during release. These complex 3D shapes can be captured by studying perturbed states of the systems in a small neighborhood of the straight configuration [13], [29], [30] (see File S1 for details). This can be systematically implemented by expanding the relevant variables 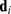 and 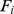 as power series in a small perturbation parameter 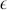


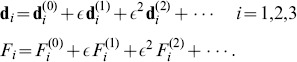
(8)Substituting Eqns. (8) into (3), the Kirchhoff equations to the 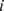-th order 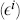 can be obtained (see File S1 for details). The first order solution is then assumed to take the form 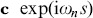, where 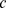 is the amplitude vector and 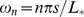 represents the angular frequency of the mode. Assuming there are no constraints on the rotation or displacement at both ends, we find that when the applied force 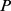 is decreased to
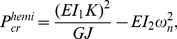
(9)a non-trivial solution to the first order equations exists. Therefore, for 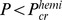 the straight configuration is unstable and complex 3D configurations are expected to grow and dominate. The shape of the modes may be obtained by solving the 2-nd order equilibrium equations, yielding
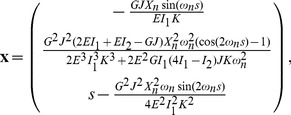
(10)where 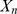 is the mode amplitude.

**Figure 6:**
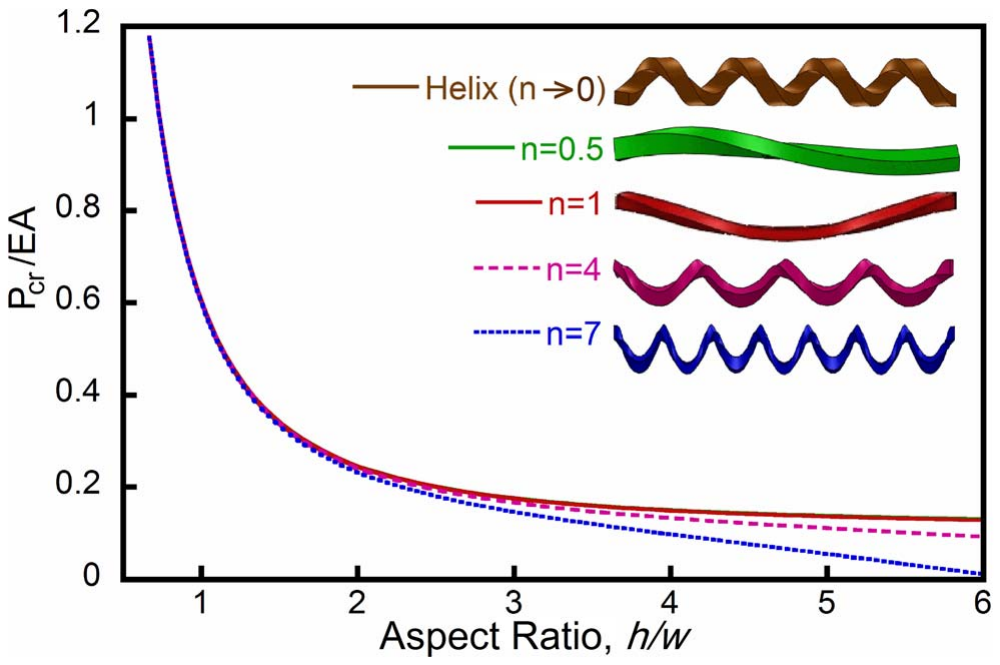
The critical loads for different buckling modes. For a small 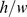 ratio, the critical end-to-end distances 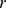 for different modes are very close to one another and difficult to distinguish. Increasing the aspect ratio by increasing the thickness decreases the critical buckling load as well as separating the individual modes. To illustrate this behavior results for four modes and the helix are shown.

The modes obtained from Eqns. 10 with 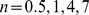 are shown in Fig. 6. Note that modes with 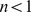 are included because the two ends of the bi-strip are allowed to rotate freely in the experiments. The modes with 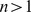 clearly resemble the hemihelices observed in the experiments and consist of multiple, periodic and alternating helical sections of opposite chiralities, separated by 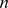 perversions. However, for 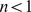 the perversion lies outside the rod, so that the system deforms into a single helical segment, leading to the formation of an helix. This is also confirmed by the fact that 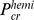 approaches 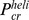 as 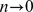. Therefore, 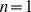 defines the boundary between forming hemihelices and helices.

In Fig. 6 we also report the evolution of the critical loads the critical loads 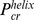 and 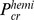 as a function of 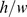 for different modes. The results clearly show that the helix is always the first to be excited. However, it is important to note that for small values of 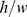 the modes are very closely spaced, while as 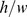 increases, the critical values for different modes become more and more separated. Therefore, for high aspect ratio bistrips, helices are more likely to form and dominate, since they evolve before hemihelical modes are triggered. In contrast, for low values of 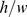 we do not expect to observe helices, since multiple modes are triggered almost simultaneously.

### Mode selection

In this section, we determine which mode grows to dominate the shape evolution during the release process. Since the stability analysis above indicates that several competing modes could form almost simultaneously for low values of 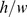, we expect not only geometric non-linearities, but also the interactions between different modes to play a role in the mode selection process, making a rigorous mathematical analysis intractable.

For this reason and to capture the instability beyond its onset we make the ad-hoc assumption that it is the fastest growing perturbation mode at the onset of the instability that will dominate the shape evolution. Although this approach neglects the contribution of geometric non-linearities and possible interactions between different modes, it has already been successfully used not only to determine the mode selected by slender rods [29], [31], [32] under a number of different loading conditions, but also to identify the spatial fluctuations that will grow in theories of phase separation, for instance [33]. Moreover, we will show that the predictions obtained using this simple analysis nicely agree with both our experimental and numerical results.

Specifically, we assume that perturbations in the shape take the form 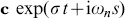 [13], [30], [34]–[36], where 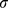 represents the growth rate of the mode (see File S1 for details). We then calculate the growth rate 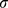 for a given value of 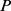 and 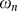 by substituting the solution into the Kirchhoff equations to the first order. When 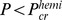 solutions with positive real values of 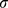 are found, identifying those perturbations that grow exponentially with time. We expect the mode with the highest growth rate 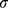 to dominate the shape evolution and to be the one observed in the experiments. In contrast, for 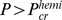 solutions with imaginary or negative 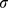 are obtained; these will be of the order of the perturbation itself, cannot grow and hence will not be observed. Finally, when 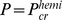 we find that 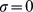 and the solution reduces to the one determined analytically in the stability analysis above.

**Figure 7:**
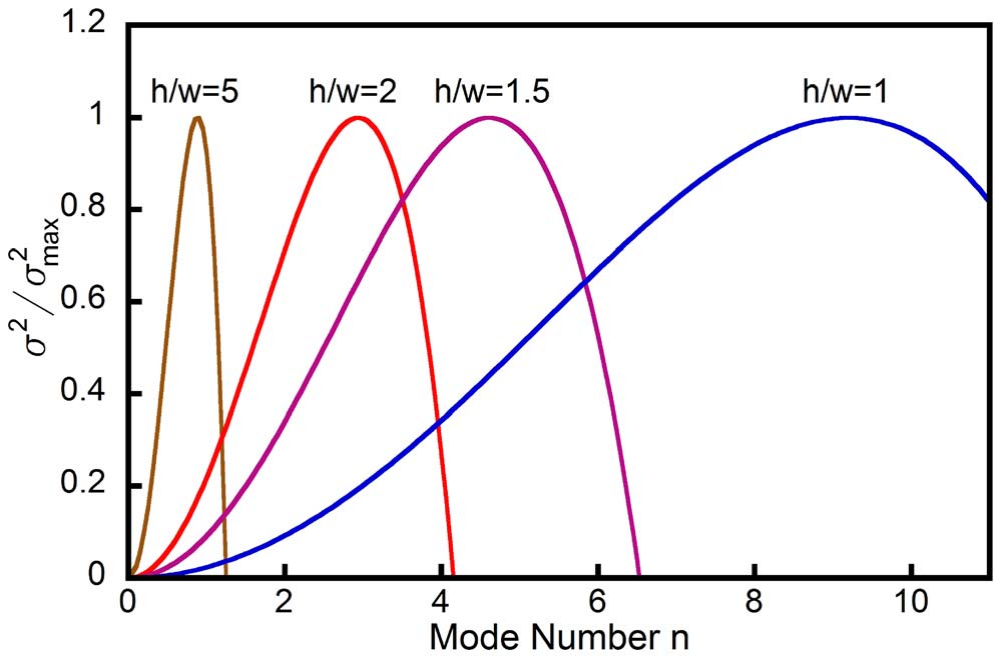
Growth rate 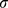 as a function of the mode number 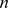 for three different strips characterized by 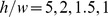, 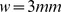, 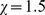 and 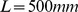. The growth rate is determined when the applied force decreases to 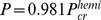.

In Fig. 7 we show the growth rate as a function of the mode number 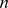 for strips with different aspect ratios 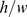. The results clearly show that the fastest growing mode in a strip with 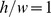 is characterized by 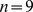. In contrast, for a higher aspect ratio strip with 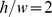 the mode with 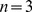 is the fastest to evolve and is expected to dominate. Finally, if 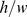 further increases, the growth rate is maximum for 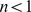, so that the formation of a helix is expected. These analytical predictions are fully consistent with the experimental results shown in Fig. 3, where it is clearly shown that the mode number monotonically decreases as a function of 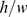.

**Figure 8:**
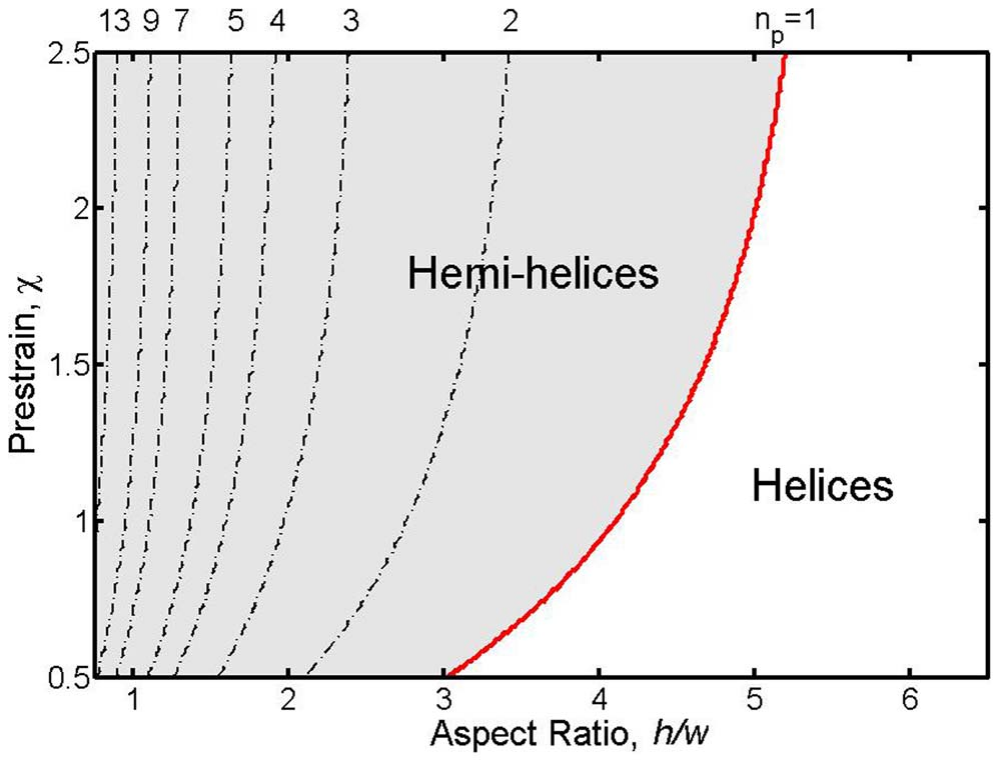
Contour plots showing the value of 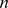 for which the growth rate is maximum as function to 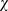 and 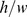. The growth rates are calculated for 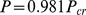. Black dotted lines show the boundaries between modes with different number of perversions 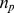, while the red line corresponds to 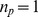 and separates hemihelices (on its left) from helices (on its right). For clarity not all the higher modes are shown.

Next we identify the boundary delineating the formation of hemihelices and helices. We find the mode 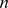 that has the maximum growth rate for rods characterized by different values of prestrain 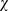 and cross-sectional aspect ratio 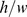. The results are reported in Fig. 8 as contour map. The dashed lines indicate the values of 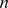 for which the growth rate is maximum and therefore corresponds to the expected number of perversions 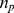. This parametric study reveals that the number of perversions in the rod after bifurcation is only moderately affected by the pre-strain 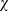, while the aspect ratio 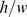 is found to have a significant effect, again consistent with experiment. In particular, the red line in the plot marks the configurations for which 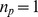. As highlighted above, if 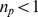 the perversion lies outside the ends of the rod, so that the system deforms into a single helical segment and the formation of helices is expected during the release process. Therefore, the red line defines the geometric transition between hemihelices and helices and the shaded region indicates where hemihelices form.

## Conclusions

Our experimental and modeling studies show that there is a well defined structural transition between the formation of a helix and a hemihelix. The helix is energetically preferred over the straight rod as the load stretching a rod is reduced (see Fig. S10 in File S1), but other buckling mode instabilities associated with twisting can intervene preventing the shape evolution from following the lowest energy path [15]. Instead, these instability modes result in the formation of hemihelices with multiple perversions even though their total energies are higher than the simple helix [9], [13], [15]. We find that the growth of the buckling mode instabilities depends principally on the aspect ratio of the rod cross-section with the fastest growing mode determing the number of perversions formed. Rods with a high-aspect ratio are less susceptible to twisting instabilities and so form helices. The perversions once formed are trapped in higher energy states and can only be removed by the application of an external set of forces, for instance rotating one end with respect to the other. Additional perversions can also be introduced by counter-rotation of the ends as is common experience with winding and unwinding telephone cords.

Our analysis correctly captures both the trend of perversion number with aspect ratio as well as the hemihelix to helix transition as represented on the phase diagram of Fig. 8 and found experimentally Fig. 3. The fact that the same geometrical features are predicted to form in either linear or nonlinear elastic materials, as borne out by finite element simulations [15], indicates that while the actual number of perversions may differ and the transition from the hemihelix to helix may occur at a somewhat different aspect ratio for different materials (see Fig. S9 in File S1), the formation of hemihelices is not dependent on the material having a specific constitutive deformation behavior. It is essential, though, that the material be capable of large strains without failing. Indeed, it is highly probable that the reason hemihelices with multiple perversions have escaped notice in the past has been that most man-made materials, unlike elastomers, would fracture well before these strains could be achieved.

We note from finite element simulations in our earlier work [15] that perversions have an elastic self-energy. This leads to the perversions repelling one another and adopting a regular spacing. Indeed, observations of the stretched bi-strip upon release, some of which are shown in videos in the supplemental information, reveal complex transients associated with the perversions. These include re-adjustment of their positions during release as well as the initial formation of a single perversion at one end that then moves along the bi-strip to the other end where it vanishes.

In a wider context, the emergence of intricate and well controlled patterns in natural slender structures, such as flowers or leaves, is often the result of specific mechanical instabilities. However, at present, our understanding of how in-plane stresses generated by nonuniform growth lead to such 3D complex shapes is incomplete. Furthermore, there is a need for translating these rules into simple strategies to engineer flat systems that shape themselves into desired 3D configurations. Indeed, the original motivation for this work was to understand which 3D shapes could be produced from flat elastic strips using one particular set of simple stretching and joining operations. Much to our surprise, we discovered that a wide range of possible shapes can be attained in our simple stressed system, specifically hemihelices with multiple chirality-reversing perversions formed under certain conditions rather than the simple helix we had expected. In summary, this work has shown, experimentally and through analysis, that by carefully controlling the cross-sectional aspect ratio and the pre-strain, it is possible to form a helix or a hemihelix with a prescribed number of perversions. We believe that our findings hold promise for fully deterministic manufacture of three-dimensional objects from pre-strained flat parts.

## Materials and Methods

### Materials

The elastomer strips were cut from silicone rubber sheets formed by casting a two-part commercial product (Dragon Skin 10 Slow, Reynolds Advanced Materials), between two large parallel acrylic sheets (20×60 cm) held 3 mm apart. Coloring agents (Silicone pigment, Reynolds Advanced Materials) were added before mixing. After curing for 7 hours at room temperature, the top acrylic sheet was peeled away and then the strips were cut to the desired width using a blade and peeled away from the bottom acrylic sheet. The glue was also a silicone rubber product (Sil-Poxy, Reynolds Advanced Materials).

### Unloading procedure

The experimental observations of the unloading of the bi-strip were made under axial loading and free-rotation conditions. This was achieved by attaching the bi-strips between two thin nylon fibers, one attached to a fixed frame and the other to a weighted container free to rotate (Fig. S1 in File S1). The container, which had a small hole in the bottom, was filled with small metal balls to stretch the bistrip. As the metal balls ran out of the hole in the container, the gravitational force on the bi-strip steadily decreased and the deformation of the bistrip was recorded (Videos S1, S2, and S3, Figs. S2, S3, and S4 in File S1). Similar experiments but with neither end permitted to rotate (Videos S4, S5, and S6) and only one end permitted to rotate (Video S7, Fig. S6 in File S1) were also performed. Unloading experiments were also performed under water to dampen vibrations and oscillations (Video S8).

### Simulations

The commercial FE software Abaqus FEA was used for the analysis, employing the the Abaqus/Explicit solver. Three-dimensional models were built using 3D linear reduced integration elements (ABAQUS element type C3D8R). The accuracy of each mesh was ascertained through a mesh refinement study. Dynamic explicit simulations were performed and quasi-static conditions were ensured by monitoring the kinetic energy and introducing a small damping factor. The analysis were performed under force control. The material model was implemented into Abaqus/Explicit through user defined subroutine VUMAT. The material response was captured using the hyper-elastic Gent model [22]. More details on the FE simulations are provided in the File S1.

## Supporting Information

File S1 **Details for experimental set-up, finite element simulations and analytical model. This file also contains Figures S1–S16.**
(PDF)

Video S1 **Video recording for a hemihelix with multiple perversions.** Both ends are free to rotate.
(WMV)

Video S2 **Video recording for a hemihelix with only one perversion.** Both ends are free to rotate.
(WMV)

Video S3 **Video recording for a helix.** Both ends are free to rotate.
(WMV)

Video S4 **Video recording for a hemihelix with multiple perversions.** Neither end is free to rotate. The geometry and prestretch are the same as those in Video S1.
(WMV)

Video S5 **Video recording for a hemihelix with two perversions.** Neither end is free to rotate. The geometry and prestretch are the same as those in Video S2.
(WMV)

Video S6 **Video recording for a hemihelix with one perversion.** Neither end is free to rotate. The geometry and prestretch are the same as those in Video S3.
(WMV)

Video S7 **Video recording for a helix.** One end is free to rotate while the other is fixed. The geometry and prestretch are the same as those in Video S3.
(WMV)

Video S8 **Video recording for a hemihelix with one perversion under water.** Both ends are free to rotate. The geometry and prestretch are the same as those in Video S2.
(WMV)
