# Correction: Structural Transition from Helices to Hemihelices

**DOI:** 10.1371/journal.pone.0139525

**Published:** 2015-09-25

**Authors:** Jia Liu, Jiangshui Huang, Tianxiang Su, Katia Bertoldi, David R. Clarke

The authors would to like to add a citation to a previously published article on a similar topic:

Domokos, Gábor, and Timothy J. Healey. "Multiple helical perversions of finite, intrinsically curved rods." International Journal of Bifurcation and Chaos 15.03 (2005): 871–890.

This work relates to the present paper in the following way: the authors numerically study the response of a uniform rod of finite length with intrinsic curvature and identify equilibria characterized by an arbitrary number of perversions with intermittent helical segments.
