# Supplementary material for: Structural Transition from Helices to Hemihelices
Source: PLoS One. 2014 Apr 23;9(4):e93183. doi: 10.1371/journal.pone.0093183 (PMC3997338; doi:10.1371/journal.pone.0093183)
Supplement: File S1 — Details for experimental set-up, finite element simulations and analytical model. This file also contains Figures S1–S16. (PDF) [file pone.0093183.s001.pdf]

Supplementary Material for the article "From helices to hemihelices"

Jia Liu[+,1], Jiangshui Huang[+,1], Tianxiang Su[+], Katia Bertoldi[+,*,2], and David R. Clarke[+,2]

[+]*School of Engineering and Applied Sciences, Harvard University, Cambridge, MA 02138*
[*]*Kavli Institute, Harvard University, Cambridge, MA 02138*
[1]*Contributed equally to this work*
[2]*To whom correspondence may be addressed. E-mail: bertoldi@seas.harvard.edu and clarke@seas.harvard.edu*

We present three sets of supplemental materials:

S1 Description of the experimental arrangement and sequences of images captured from video recordings of the unloading of stretched bi-strips. Details are given in the figure captions.

S2 Details of the finite element simulations used to produce the simulation results presented in the text.

S3 Full details of the Kirchhoff analysis as well as the stability and instability to perturbations that leads to the analytical findings presented in the text.

S1 Experimental loading arrangement, video sequences and phase diagram obtained under non-rotating end conditions

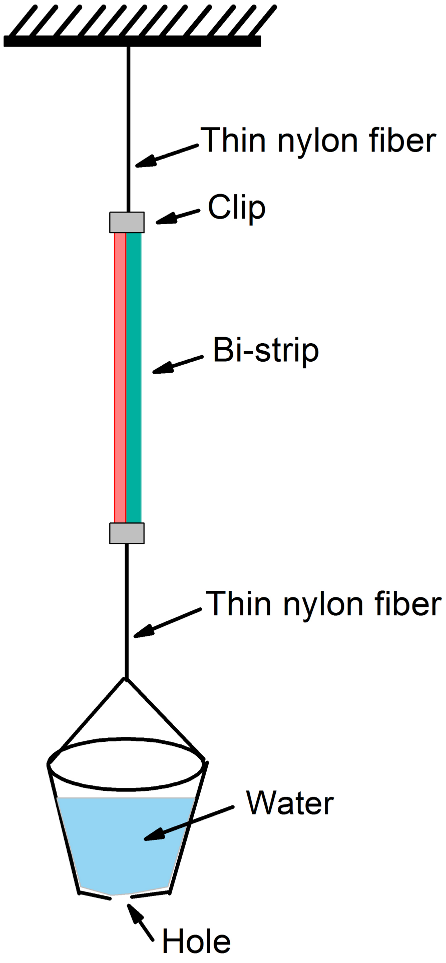

Figure S1: Experimental configuration used to alter the force stretching the bi-strip while allowing both ends of the bi-strip to freely rotate. The gravitational force on the weight attached to the bottom of the bi-strip stretches it. The weight consists of a container filled with either water or ball bearings. As the water or metal balls flow out of the container, the gravitational force applied to the bi-strip through the nylon fiber gradually decreases. The same configuration, with metal balls, was used to perform some experiments in water rather than in air to dampen transients.

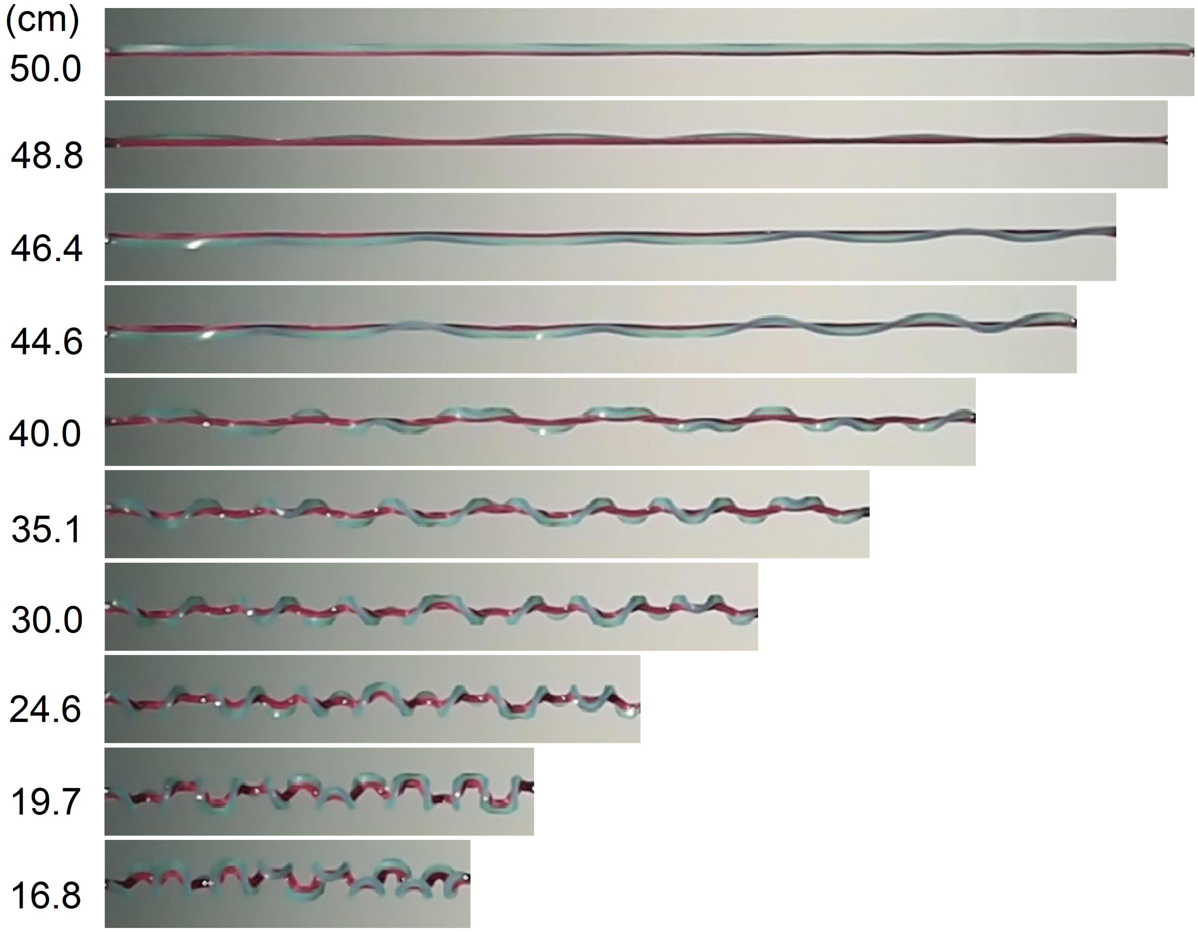

Figure S2: Sequence of images as the stretching force applied to the ends of a bi-strip is slowly reduced showing the formation of a hemihelix with eleven perversions. Width w=3mm, thickness h=2.5mm, length L =50 cm, and χ = 1.5 (Video S1). The distance between the two ends of the bi-strip indicated at the side of each image, decreases as the force is decreased. Perversions appear to form immediately unloading commences and while their amplitude steadily increases, their number remains unchanged.

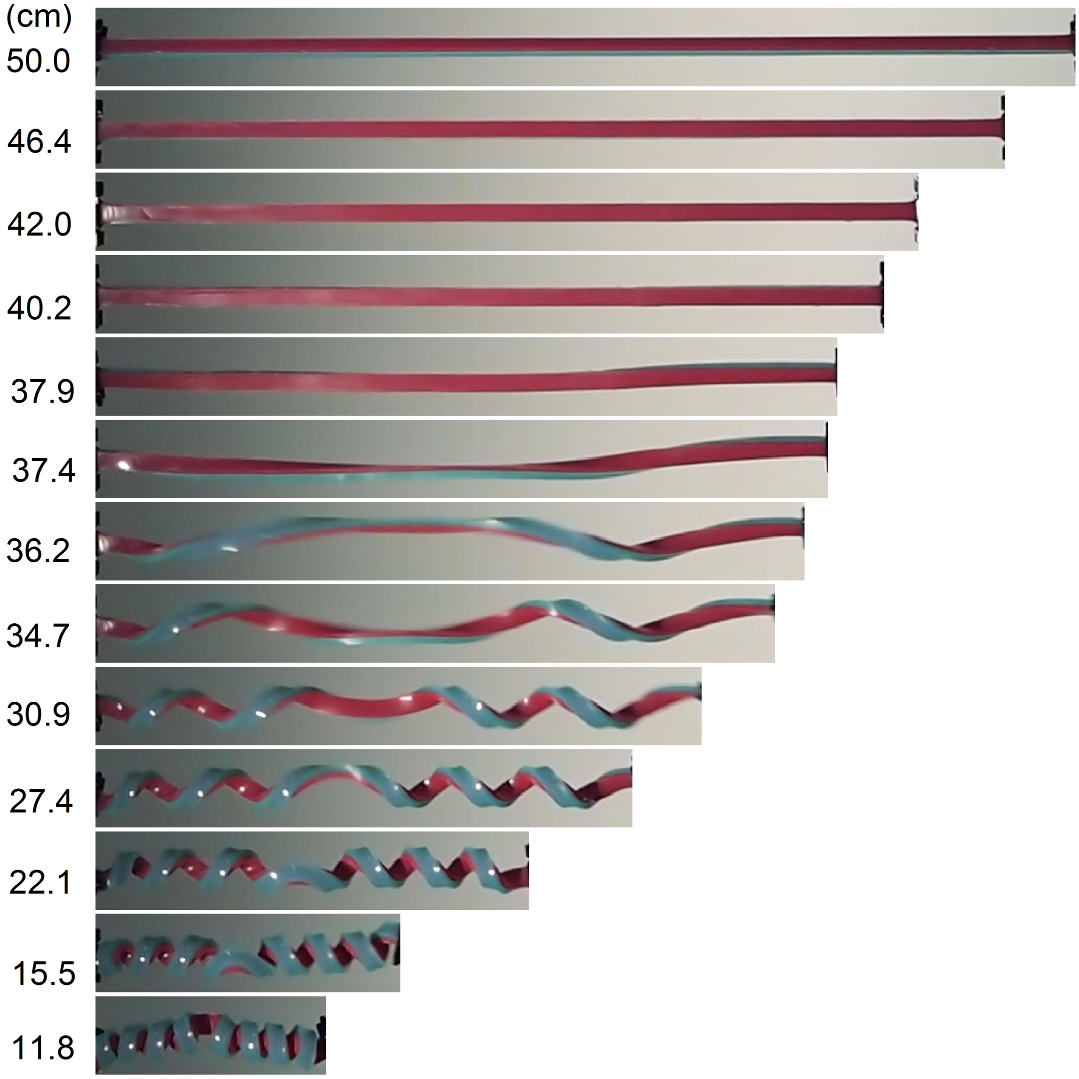

Figure S3: Similar images to those in figure S2 for a bistrip of the same dimensions except that the thickness is h=8mm (Video S2). As the distance between the two ends decreases, the bi-strip initially remains straight and flat but then a single perversion begins to form in the middle and grows to separate two helices of opposite chirality.

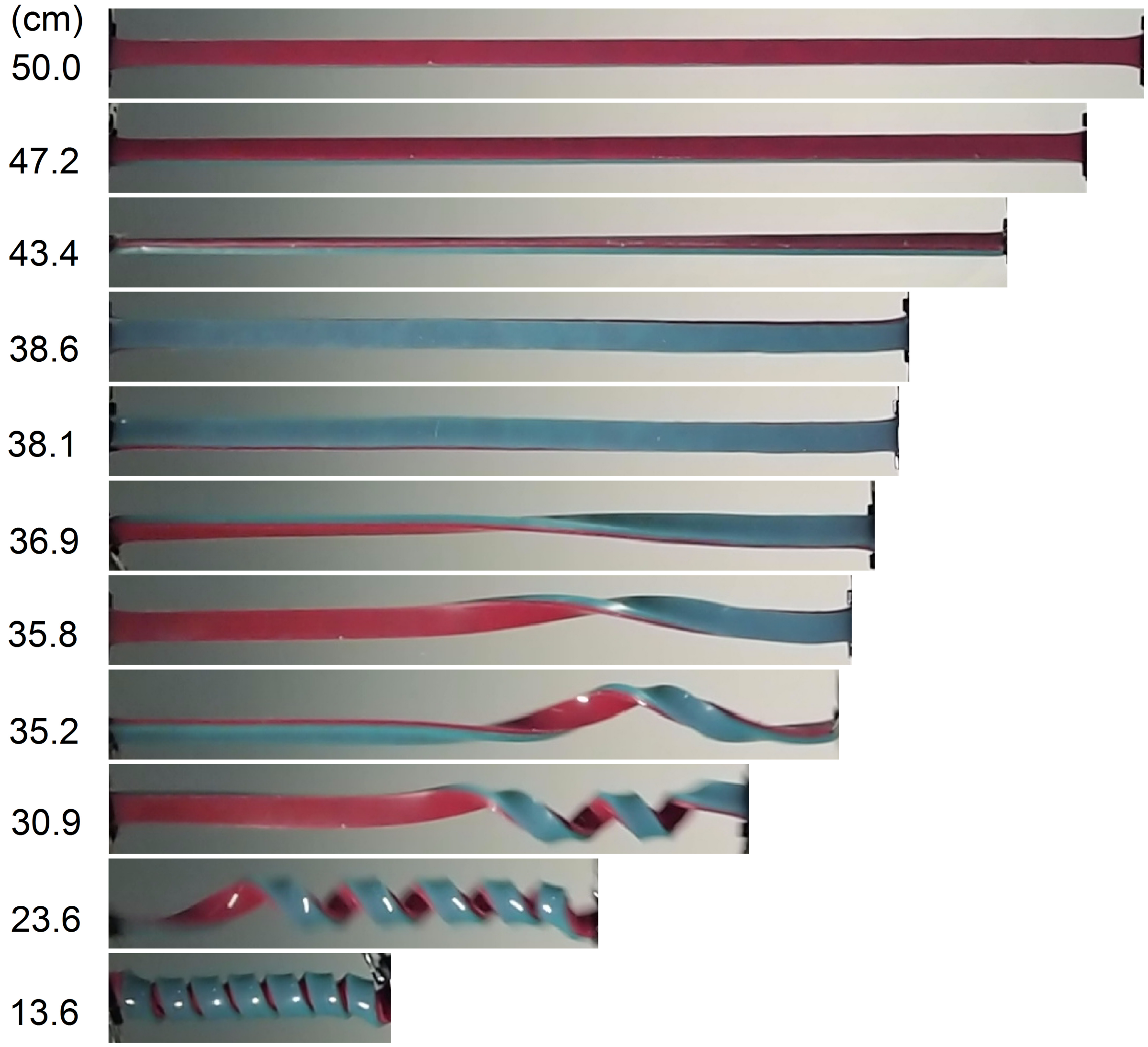

Figure S4: A sequence of images illustrating the formation of a simple helix upon unloading. (Video S3). Images of the unloading of a similar strip as in figure S2 and S3 except the strip had a greater thickness, h = 12 mm. At first, although the bi-strip rotates about its long axis, it remains straight and flat. As the distance is decreased further, an initial twist develops near the right-hand end, grows and then propagates along the bi-strip to form a simple helix.

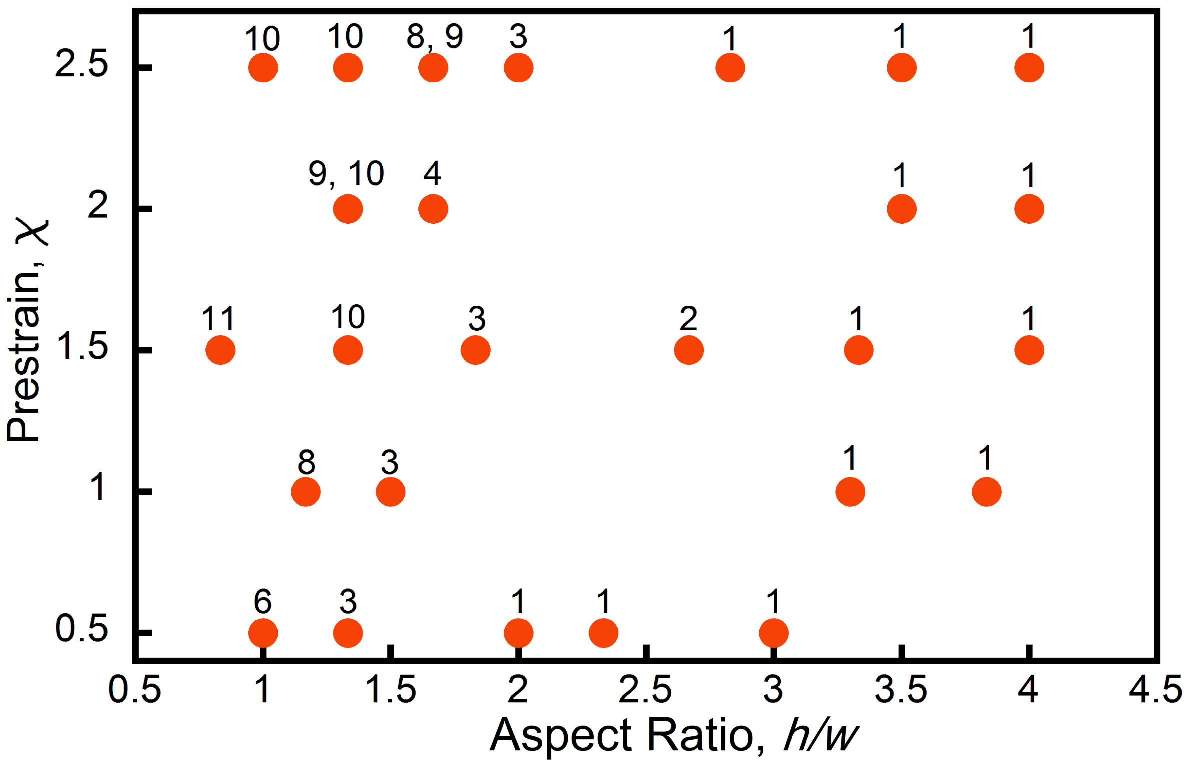

Figure S5: Phase diagram obtained when both ends of the bi-strip are clamped to prevent them from rotating. Compared to the phase diagram when the ends are free to rotate, the main difference is that helices do not form. Also, the number of perversions formed can be slightly different. For example, for h/w = 2.67 and $\chi = 1.5$, there is only one perversion when the ends can rotate but two perversions when they are clamped. The length, L, is 50 cm for each bi-strip.

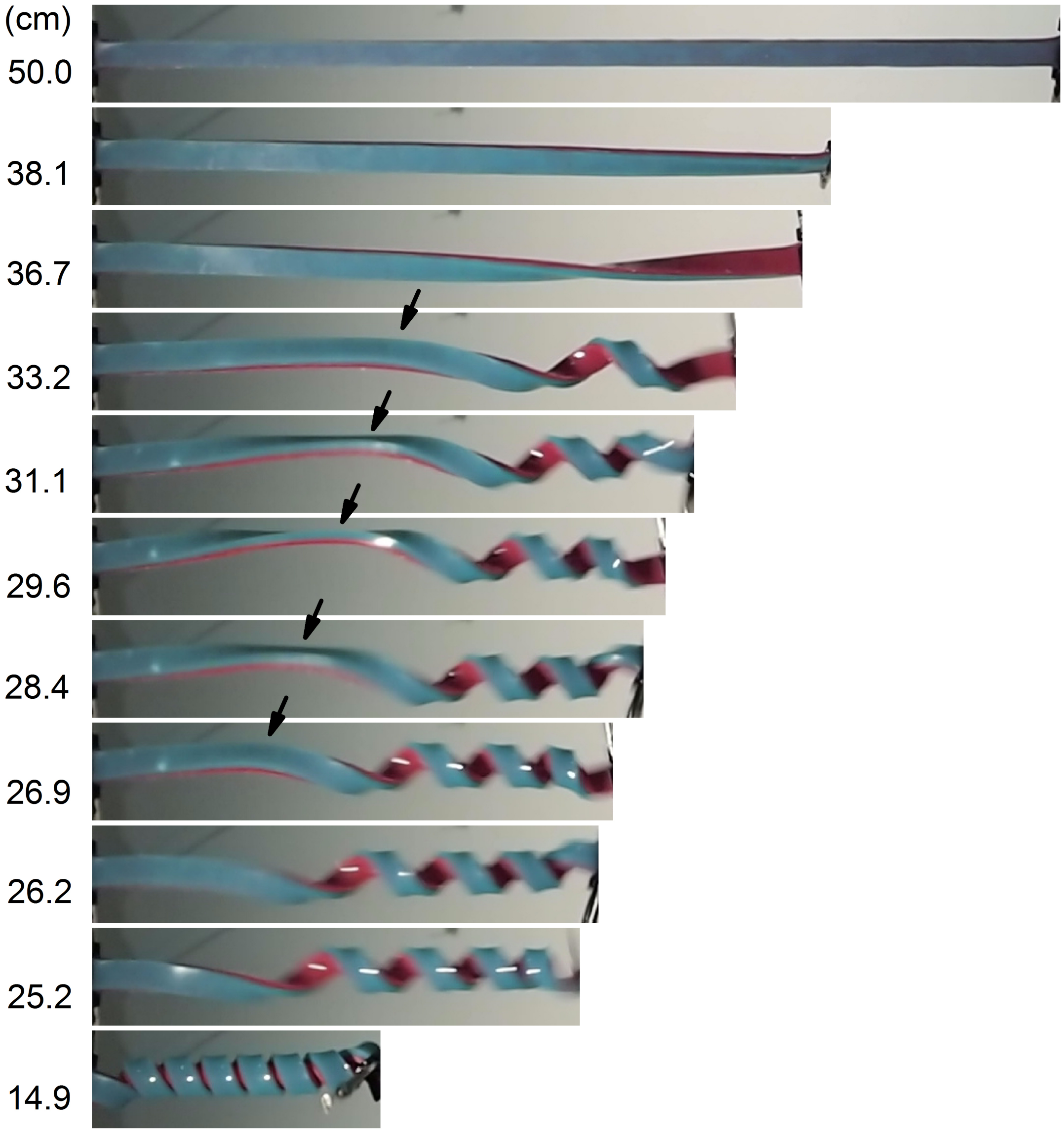

Figure S6: Images of the unloading of the same strip as in figure S4, but under a fixed boundary condition at the left end and free to rotate at the right end end (Video S7). In the process of forming a helix, a single perversion, marked by the arrow, begins to form but is then eliminated.

S2 Finite Element Simulations

To study the formation of the helices and hemi-helices and the conditions under which they form, the combination of twisting, compression and bending, together with the highly nonlinear constitutive behavior of elastomer need to be included. To simulate this complicated behavior, we first use finite element numerical simulations. Before describing these simulations in detail, the constitutive behavior of the elastomer is presented.

S2.1 The material model

Uniaxial tensile stress-strain tests performed on the bulk material show that it exhibits a large strain elastic behavior typical of elastomers with significant stiffening [1]. To capture the observed deformation response we modeled the material as a hyper-elastic solid and computed the stresses and elastic energies using the nearly-incompressible Gent model [10]. Here we briefly review the mathematical description of the model as well as basic concepts in the theory of large deformation.

Formally, we denote $\mathbf{X}$ as the position vector of a material point in the undeformed configuration. During deformation, the material point gains a new position, which is marked by $\mathbf{x}$. The mapping matrix $\mathbf{F} = \partial\mathbf{x}/\partial\mathbf{X}$, named the deformation gradient, therefore connects the undeformed and deformed states. The determinant of the matrix $J = \det(\mathbf{F})$ represents the local volume change of the bulk material. For an isotropic hyperelastic material, the strain energy density W, is a function of the invariants of the left Cauchy-Green tensor $\mathbf{B} = \mathbf{F}\mathbf{F}^T$:

$$W = W(I_1, I_2, I_3), \tag{S1}$$

where

$$I_1 = \mathrm{tr}(\mathbf{B}),\ I_2 = [(\mathrm{tr}\mathbf{B})^2 - \mathrm{tr}\mathbf{B}^2],\ I_3 = \det(\mathbf{B}) = J^2. \tag{S2}$$

Consequently, the Cauchy stress, a measure of the force acting on an element of area in the deformed material, is given by:

$$\boldsymbol{\sigma} = \frac{2}{J}\frac{\partial W}{\partial I_1}\mathbf{B} + \frac{\partial W}{\partial J}\mathbf{I}, \tag{S3}$$

where $\mathbf{I}$ is the identity tensor.

Particularly, for the Gent model, the strain energy density is,

$$W = -\frac{\mu}{2}J_m \ln\left(1 - \frac{I_1 - 3}{J_m}\right) - \mu \ln J + \left(\frac{K}{2} - \frac{\mu}{J_m}\right)(J-1)^2, \tag{S4}$$

where μ and K are the initial shear and bulk moduli, respectively, and J_m is a parameter related with the strain saturation. Eqn. (S3) then suggests the Cauchy stress is:

$$\boldsymbol{\sigma} = \frac{\mu J_m}{J(J_m - I_1 + 3)}\mathbf{B} - \frac{\mu}{J}\mathbf{I} + \left(K - \frac{2\mu}{J_m}\right)(J-1)\mathbf{I}. \tag{S5}$$

Additionally, we can obtain the nominal stress $\mathbf{s} = J\boldsymbol{\sigma}\cdot\mathbf{F}^{-T}$, which measures the force acting on an element of area in the undeformed configuration,

$$\mathbf{s} = \frac{\mu J_m}{J_m - I_1 + 3}\mathbf{F} - \mu\mathbf{F}^{-T} + \left(K - \frac{2\mu}{J_m}\right)J(J-1)\mathbf{F}^{-T}. \tag{S6}$$

The parameters μ, K, and J_m are obtained by fitting to the experimental tensile load-displacement curves, yielding values $\mu = 0.06$MPa, $K = 24$MPa and $J_m = 28.2$ [1].

To account for the prestrain ratio χ, we decompose the deformation gradient into a load-induced part $\mathbf{F}^L$ and a prestretch induced part $\mathbf{F}^S$ following the multiplicative decomposition method originally introduced by Kroner and Lee [11, 12],

$$\mathbf{F} = \mathbf{F}^L \mathbf{F}^S, \text{ with } \mathbf{F}^S = \text{diag}(\chi + 1, 1/\sqrt{\chi + 1}, 1/\sqrt{\chi + 1}). \tag{S7}$$

S2.2 Numerical Analysis

The commercial FE software Abaqus FEA was used for the analysis, employing the the Abaqus/Explicit solver. The material model was implemented into Abaqus/Explicit through user defined subroutine VUMAT. Three-dimensional models are built using 3D linear reduced integration elements (ABAQUS element type C3D8R). The accuracy of each mesh was ascertained through a mesh refinement study. Thereafter, dynamic explicit simulations were performed and quasi-static conditions were ensured by monitoring the kinetic energy and introducing a small damping factor. The analyses were performed under force control.

S3 Analytical Model

S3.1 The Kirchhoff model

The Kirchhoff model provides a well-established framework to study the statics and dynamics of elastic rods [13, 14]. In this section we introduce the basic notations, briefly review the derivation of the Kirchhoff equations and finally summarize how the stability of a rod can be investigated by studying perturbed states of the system.

S3.1.1 Kinematics

We consider an inextensible and unshearable rod in the 3D space [3, 7, 8], whose centerline is described by a position vector $\mathbf{x}(s, t)$, where s is the arc-length and t is the time (see Fig. S7). Note that due to the inextensible assumption, the total length L of the rod does not change during deformation, so that $s \in [0,\, L]$. In addition, a local director basis

$$(\mathbf{d}_1,\, \mathbf{d}_2,\, \mathbf{d}_3) = (\mathbf{d}_1(s,\, t)),\, \mathbf{d}_2(s,\, t),\, \mathbf{d}_3(s,\, t)), \tag{S8}$$

is associated to the rod and the vector $\mathbf{d}_3$ is identified as the unit tangent vector to the curve,

$$\mathbf{d}_3 \equiv \mathbf{x}', \tag{S9}$$

where here and in what follows $(\cdot)' = \partial(\cdot)/\partial s$. Furthermore, $\mathbf{d}_1$ and $\mathbf{d}_2$ are two unit vectors in the plane normal to $\mathbf{d}_3$, so that $(\mathbf{d}_1,\, \mathbf{d}_2,\, \mathbf{d}_3)$ forms a right-handed triad (i.e. $\mathbf{d}_2 = \mathbf{d}_3 \times \mathbf{d}_1$, $\mathbf{d}_1 = \mathbf{d}_2 \times \mathbf{d}_3$). For the sake of simplicity, $\mathbf{d}_1$ and $\mathbf{d}_2$ are chosen to lie along the principal axes of inertia of the cross-section.

The condition of orthonormality implies the existence of a twist vector $\boldsymbol{\kappa} = \kappa_1 \mathbf{d}_1 + \kappa_2 \mathbf{d}_2 + \kappa_3 \mathbf{d}_3$ satisfying

$$\mathbf{d}_i' = \boldsymbol{\kappa} \times \mathbf{d}_i, \quad i = 1, 2, 3, \tag{S10}$$

so that

$$\mathbf{d}_1' = -\kappa_2 \mathbf{d}_3 + \kappa_3 \mathbf{d}_2, \;\; \mathbf{d}_2' = \kappa_1 \mathbf{d}_3 - \kappa_3 \mathbf{d}_1, \;\; \mathbf{d}_3' = -\kappa_1 \mathbf{d}_2 + \kappa_2 \mathbf{d}_1. \tag{S11}$$

κ_1 and κ_2 are called material curvatures and express how much the frame $(\mathbf{d}_1,\, \mathbf{d}_2,\, \mathbf{d}_3)$ rotates about the directions $\mathbf{d}_1$ and $\mathbf{d}_2$ of the cross section. In contrast, κ_3 is called the twist density and expresses how much the director frame rotates about the direction $\mathbf{d}_3$.

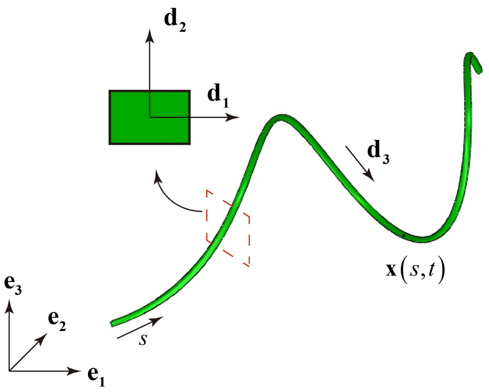

Figure S7: The configuration of an elastic rod is represented by a curve $\mathbf{x}(s,t)$ and by a local director basis ($\mathbf{d}_1$, $\mathbf{d}_2$, $\mathbf{d}_3$).

S3.1.2 Dynamics

In Kirchhoff's theory of elastic rods all physically relevant stresses are considered as cross-sectional averages at each point along the axis of the rod. Kirchhoff equations relate the resultant force $\mathbf{F}$ and moment $\mathbf{M}$ acting on the cross section to the director basis.

Let $\mathbf{p} = \mathbf{p}(s,\, X_1,\, X_2,\, t)$ be the Piola stress vector, where X_1 and X_2 are the coordinates along the principal directions on the cross-section. The Piola stress vector $\mathbf{p}$ gives the density of the elastic forces exerted on the material surface $\Omega(s)$ by the material on the side of increasing s at the point with material coordinates (s, X_1, X_2). The resultant force acting on the cross section $\Omega(s)$ is then given by

$$\mathbf{F} = \mathbf{F}(s,t) = \iint_{\Omega(s)} \mathbf{p}\, \mathrm{d}X_1 \mathrm{d}X_2. \tag{S12}$$

Similarly, the resultant moment about $\mathbf{x}(s,t)$ of the stresses acting on $\Omega(s)$ is

$$\mathbf{M} = \mathbf{M}(s,t) = \iint_{\Omega(s)} \mathbf{r} \times \mathbf{p}\, \mathrm{d}X_1 \mathrm{d}X_2, \tag{S13}$$

where the vector $\mathbf{r}$ is defined so that

$$\mathbf{y}(s, X_1, X_2, t) = \mathbf{x}(s,t) + \mathbf{r}(s, X_1, X_2, t), \tag{S14}$$

with $\mathbf{y}$ denoting the position vector of an arbitrary material point on the cross-section.

When no external forces/moments are applied, the balance laws for linear and angular momentum yield

$$\frac{\mathrm{d}\mathbf{F}}{\mathrm{d}s} = \iint_{\Omega(s)} \rho\, \ddot{\mathbf{y}}\, \mathrm{d}X_1 \mathrm{d}X_2, \tag{S15}$$

$$\frac{\mathrm{d}\mathbf{M}}{\mathrm{d}s} + \frac{\mathrm{d}\mathbf{x}}{\mathrm{d}s} \times \mathbf{F} = \iint_{\Omega(s)} \rho\, \mathbf{r} \times \ddot{\mathbf{y}}\, \mathrm{d}X_1 \mathrm{d}X_2,$$

where $\dot{(\cdot)} = \partial(\cdot)/\partial t$ and ρ is the density (mass per unit volume) that is taken to be constant during deformation. Since for an unshearable rod (i.e. a rod for whose cross-section only undergoes rigid body motion during deformation) the position of a material point $\mathbf{y}$ on the cross-section is given by

$$\mathbf{y}(s, X_1, X_2, t) = \mathbf{x}(s,t) + \mathbf{r}(s, X_1, X_2, t) = \mathbf{x}(s,t) + X_1 \mathbf{d}_1(s,t) + X_2 \mathbf{d}_2(s,t), \tag{S16}$$

Eqns. (S15) become

$$\begin{aligned}\mathbf{F}' &= \rho\, A\, \ddot{\mathbf{x}}, &\text{(S17)}\\ \mathbf{M}' + \mathbf{d}_3 \times \mathbf{F} &= \rho\left(I_2 \mathbf{d}_1 \times \ddot{\mathbf{d}}_1 + I_1 \mathbf{d}_2 \times \ddot{\mathbf{d}}_2\right), &\text{(S18)}\end{aligned}$$

where A is the cross-sectional area and I_1 and I_2 are the principal moments of inertial of the cross section. Note that after differentiation with respect to s and use of Eqn. (S9), Eqn. (S17) can be rewritten as

$$\mathbf{F}'' = \rho\, A\, \ddot{\mathbf{d}}_3. \tag{S19}$$

The Kirchhoff equations are then completed by the linear constitutive relation

$$\mathbf{M} = E\, I_1(\kappa_1 - \kappa_1^{(u)})\mathbf{d}_1 + E\, I_2(\kappa_2 - \kappa_2^{(u)})\mathbf{d}_2 + G\, J(\kappa_3 - \kappa_3^{(u)})\mathbf{d}_3, \tag{S20}$$

where J is the torsion constant which depends on the cross sectional shape, E and G are the Young's and shear modulus of the material, respectively. Moreover, in Eqn. (S20) we have introduced the intrinsic curvature $\boldsymbol{\kappa}^{(u)} = \kappa_1^{(u)}\mathbf{d}_1 + \kappa_2^{(u)}\mathbf{d}_2 + \kappa_3^{(u)}\mathbf{d}_3$ to describe a rod which in its unstressed state has a locally non-vanishing curvature. Finally, we note that the torsion constant J is defined as

$$J = \iint_{\Omega(s)} \left(X_1^2 + X_2^2 + X_1 \frac{\partial \Phi}{\partial X_2} - X_2 \frac{\partial \Phi}{\partial X_1}\right) \mathrm{d}X_1 \mathrm{d}X_2, \tag{S21}$$

where Φ is the warping function for torsion given by the linear theory of elasticity. It has been shown that for a rectangular cross-section, J can be well approximated by [4]

$$J \approx ab^3 \left(\frac{1}{3} - 0.21 \frac{b}{a}\left(1 - \frac{b^4}{12a^4}\right)\right), \tag{S22}$$

where a and b denote the length of the long and short side of the rectangular, respectively.

S3.1.3 Statics

If the system is in static equilibrium, the right-hand sides of eqns. (S17)–(S18) are zero, so that

$$\begin{aligned}\mathbf{F}' &= 0, &\text{(S23)}\\ \mathbf{M}' + \mathbf{d}_3 \times \mathbf{F} &= 0.\end{aligned}$$

Writing the resultant force as $\mathbf{F} = F_1\mathbf{d}_1 + F_2\mathbf{d}_2 + F_3\mathbf{d}_3$ and projecting Eqns. (S23) along the director basis we obtain:

$$\begin{gathered} F_1' - F_2\kappa_3 + F_3\kappa_2 = 0, \qquad \text{(S24)}\\ F_2' - F_3\kappa_1 + F_1\kappa_3 = 0,\\ F_3' - F_1\kappa_2 + F_2\kappa_1 = 0,\\ EI_1\kappa_1' - EI_2(\kappa_2 - \kappa_2^{(u)})\kappa_3 + GJ(\kappa_3 - \kappa_3^{(u)})\kappa_2 - F_2 = 0,\\ EI_2\kappa_2' - GJ(\kappa_3 - \kappa_3^{(u)})\kappa_1 + EI_1(\kappa_1 - \kappa_1^{(u)})\kappa_3 + F_1 = 0,\\ GJ\kappa_3' - EI_1(\kappa_1 - \kappa_1^{(u)})\kappa_2 + EI_2(\kappa_2 - \kappa_2^{(u)})\kappa_1 = 0. \end{gathered}$$

S3.1.4 Stability

If the exact solution of the Kirchhoff equations is known, the stability of the configuration can be investigated by studying perturbed states of the systems in a small neighborhood of the reference solution [5, 6]. This can be systematically done by expanding the relevant variables $\mathbf{d}_i$ and F_i as power series in a small parameter ϵ, which characterizes the distance from the reference configuration,

$$\mathbf{d}_i = \mathbf{d}_i^{(0)} + \epsilon\, \mathbf{d}_i^{(1)} + \epsilon^2\, \mathbf{d}_i^{(2)} + \cdots \quad i = 1, 2, 3, \tag{S25}$$

$$F_i = F_i^{(0)} + \epsilon\, F_i^{(1)} + \epsilon^2\, F_i^{(2)} + \cdots \quad i = 1, 2, 3, \tag{S26}$$

where $\mathbf{d}_i^{(0)}$ and $F_i^{(0)}$ denote the reference (unperturbed) director basis and force components, respectively. Since the orthonormality condition requires $\mathbf{d}_i \cdot \mathbf{d}_j = \delta_{ij}$, the perturbed basis $\mathbf{d}_i^{(j)}$ can be expressed in terms of the unperturbed basis $\mathbf{d}_i^{(0)}$ as

$$\mathbf{d}_i^{(1)} = \sum_{j=1}^{3} A_{ij}^{(1)} \mathbf{d}_j^{(0)}, \tag{S27}$$

$$\mathbf{d}_i^{(2)} = \sum_{j=1}^{3} (A_{ij}^{(2)} + S_{ij}^{(2)}) \mathbf{d}_j^{(0)}, \tag{S28}$$

$$\vdots$$

$$\mathbf{d}_i^{(n)} = \sum_{j=1}^{3} (A_{ij}^{(n)} + S_{ij}^{(n)}) \mathbf{d}_j^{(0)}, \tag{S29}$$

where $\mathbf{A}^{(k)}$ is an antisymmetric matrix

$$\mathbf{A}^{(k)} = \begin{pmatrix} 0 & \alpha_3^{(k)} & -\alpha_2^{(k)} \\ -\alpha_3^{(k)} & 0 & \alpha_1^{(k)} \\ \alpha_2^{(k)} & -\alpha_1^{(k)} & 0 \end{pmatrix}. \tag{S30}$$

Furthermore, $\mathbf{S}^{(k)}$ is a symmetric matrix whose entries are only a function of $\alpha_i^{(j)}$ with $j < k$. In particular, $\mathbf{S}^{(2)}$ can be obtained explicitly as

$$\mathbf{S}^{(2)} = \frac{1}{2} \begin{pmatrix} -(\alpha_2^{(1)})^2 - (\alpha_3^{(1)})^2 & \alpha_1^{(1)}\alpha_2^{(1)} & \alpha_1^{(1)}\alpha_3^{(1)} \\ \alpha_1^{(1)}\alpha_2^{(1)} & -(\alpha_3^{(1)})^2 - (\alpha_1^{(1)})^2 & \alpha_2^{(1)}\alpha_3^{(1)} \\ \alpha_1^{(1)}\alpha_3^{(1)} & \alpha_2^{(1)}\alpha_3^{(1)} & -(\alpha_1^{(1)})^2 - (\alpha_2^{(1)})^2 \end{pmatrix}. \tag{S31}$$

We also notice that the components of the twist vector $\boldsymbol{\kappa} = \kappa_1 \mathbf{d}_1 + \kappa_2 \mathbf{d}_2 + \kappa_3 \mathbf{d}_3$ can be written in terms of the perturbed variables as [9]:

$$\mathbf{K} = \left(\mathbf{B}\, \mathbf{K}^{(0)} + \frac{\partial \mathbf{B}}{\partial s} \right) \mathbf{B}^{-1}, \tag{S32}$$

where

$$\mathbf{K} = \begin{pmatrix} 0 & \kappa_3 & -\kappa_2 \\ -\kappa_3 & 0 & \kappa_1 \\ \kappa_2 & -\kappa_1 & 0 \end{pmatrix}, \; \mathbf{K}^{(0)} = \begin{pmatrix} 0 & \kappa_3^{(0)} & -\kappa_2^{(0)} \\ -\kappa_3^{(0)} & 0 & \kappa_1^{(0)} \\ \kappa_2^{(0)} & -\kappa_1^{(0)} & 0 \end{pmatrix}, \tag{S33}$$

and

$$\mathbf{B} = \mathbf{I} + \epsilon \mathbf{A}^{(1)} + \epsilon^2 \left(\mathbf{A}^{(2)} + \mathbf{S}^{(2)}\right) + \cdots \tag{S34}$$

Substituting (S25) and (S26) into the governing equations (S18) and (S19), and defining the stationary configuration in terms of the six-dimensional vector $\boldsymbol{\mu}^{(0)} = \left(\alpha_1^{(0)}, \alpha_2^{(0)}, \alpha_3^{(0)}, F_1^{(0)}, F_2^{(0)}, F_3^{(0)}\right)$, the Kirchhoff equations to order k are obtained as

$$\begin{aligned}
O(\epsilon^0) &\;:\; \mathbf{E}(\boldsymbol{\mu}^{(0)}) = \mathbf{0}, && \text{(S35)}\\
O(\epsilon^1) &\;:\; \mathbf{L}(\boldsymbol{\mu}^{(0)}) \cdot \boldsymbol{\mu}^{(1)} = \mathbf{0}, && \text{(S36)}\\
O(\epsilon^2) &\;:\; \mathbf{L}(\boldsymbol{\mu}^{(0)}) \cdot \boldsymbol{\mu}^{(2)} = \mathbf{H}_2(\boldsymbol{\mu}^{(1)}), && \text{(S37)}\\
&\;\;\vdots
\end{aligned}$$

where $\boldsymbol{\mu}^{(k)} = \left(\alpha_1^{(k)}, \alpha_2^{(k)}, \alpha_3^{(k)}\, F_1^{(k)}, F_2^{(k)}, F_3^{(k)}\right)$, $\mathbf{E}(\boldsymbol{\mu}^{(0)}) = \mathbf{0}$ describes the trivial static solution, $\mathbf{L}(\cdot)$ is a linear operator and $\mathbf{H}_2(\cdot)$ is a quadratic operator. It is worth noting that eqns. (S35), (S36) and (S37) have to be solved sequentially.

Finally, we note that when $\boldsymbol{\mu}^{(1)}$ and $\boldsymbol{\mu}^{(2)}$ are known, the solution $\mathbf{x} = \mathbf{x}^{(0)} + \epsilon\, \mathbf{x}^{(1)} + \epsilon^2\, \mathbf{x}^{(2)}$ can be reconstructed as

$$\mathbf{x} = \int \mathbf{d}_3 \mathrm{d}s = \int \left(\mathbf{d}_3^{(0)} + \epsilon\, \mathbf{d}_3^{(1)} + \epsilon^2\, \mathbf{d}_3^{(2)}\right) \mathrm{d}s. \tag{S38}$$

In Section S3.2.3 we will show that all the 3D complex shapes observed during the release process in our experiments can be captured conducing the stability analysis outlined above.

S3.2 Modeling of pre-strained elastomeric bi-strips

This study focuses on the investigation of the complex shapes that can be produced by a simple generic process consisting of pre-straining one elastomeric strip, joining it side-by-side to another and then releasing the bi-strip. To predict such shapes analytically, the pre-strained elastomeric bi-strip is modeled as a rod with homogeneous rectangular cross section and intrinsic curvature, so that its response can be described by Kirchhoff equations. In this section we first describe how the intrinsic curvature of the equivalent rod can be obtained (Section S3.2.1), then investigate two simple equilibrium solutions to the Kirchhoff equations (Section S3.2.2) and finally study the stability of the rod (Section S3.2.3) and the mode selection process (Section S3.2.4) as the tension is slowly released.

S3.2.1 Intrinsic curvature of the equivalent rod

In the physical system considered in this study we have two strips: one non-prestretched and one prestretched. For the sake of clarity, in this section we use the subscripts a and b to denote quantities related to the non-prestretched and prestretched strips, respectively. Note that L_a, w_a and h_a correspond to L, w and h in the main text (which are the length, width and height, respectively), while L_b, w_b and h_b are denoted as L', w' and h in the main document. The two strips are different in initial length so that $L_a \neq L_b$. When the shorter strip is stretched to the length of the longer one and bonded to it, it is expected that they together will curve along the bonding layer and form an arc with angle θ and curvature K, as shown in Fig. S8(a). Therefore, the bi-strip can be modeled as a rod with homogeneous cross-section, intrinsic curvature $(\kappa_1^{(u)}, \kappa_2^{(u)}, \kappa_3^{(u)}) = (K, 0, 0)$ and length $L_* = R\,\theta$ (see Fig. S8(b)), with R denoting the radius from the center of the arc to the interface of the two bonded strips. Now we determine

the curvature K and the length L_* as functions of the dimensions and material properties of the two strips.

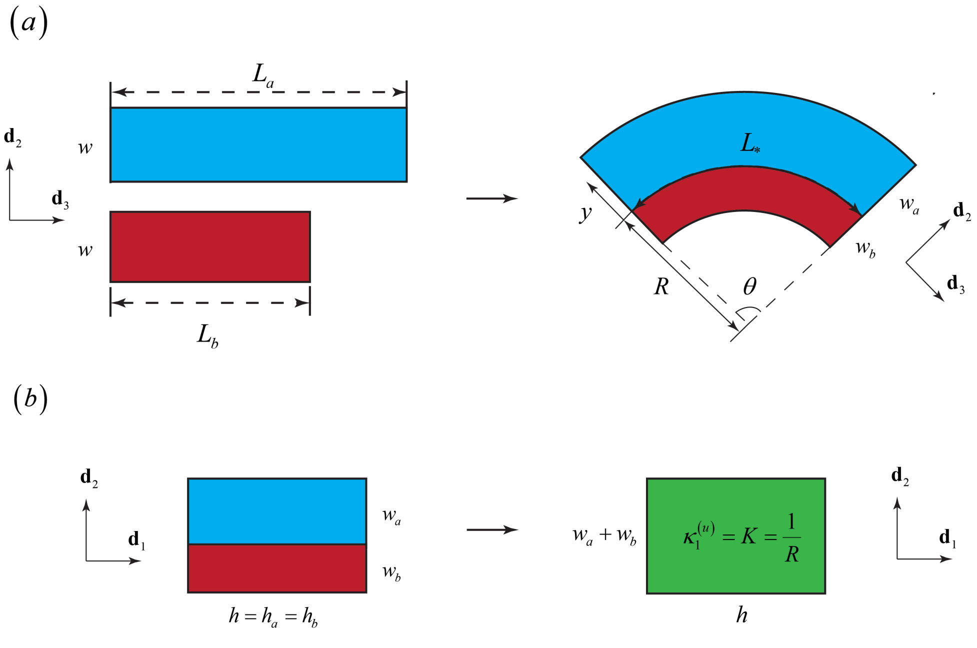

Figure S8: (a) The pre-straining operation used to form the bi-strip. Strip b is stretched until it has the same length as strip a. Then two strips are then glued together and curve along the bonding layer, forming an arc with angle θ, curvature K and length L_*. (b) The bi-strip is modeled as a rod with homogeneous cross-section, intrinsic curvature $(\kappa_1^{(u)}, \kappa_2^{(u)}, \kappa_3^{(u)}) = (K, 0, 0)$ and length $L_* = R\theta$.

Let y denote the width coordinate with $y = 0$ at the interface of the two bonded strips. According to the elastic beam theory and the assumption of pure bending, the longitudinal strain ϵ at an arbitrary point on the cross section of the two strips is given by

$$\epsilon = \begin{cases} \dfrac{(R+y)\theta - L_a}{L_a} = \dfrac{L_* + y\theta}{L_a} - 1, & y > 0 \text{ for strip } a, \\[2ex] \dfrac{(R+y)\theta - L_b}{L_b} = \dfrac{L_* + y\theta}{L_b} - 1, & y < 0 \text{ for strip } b. \end{cases} \tag{S39}$$

This deformation will result in a uniaxial state of stress, where only the normal stress σ along the longitudinal direction is non-vanishing. Mechanical equilibrium requires that the net forces and moments are zero, yielding

$$\int \sigma dA = \int_0^{w_a} \sigma_a h_a \, dy + \int_{-w_b}^{0} \sigma_b h_b \, dy = 0, \tag{S40}$$
$$\int \sigma y dA = \int_0^{w_a} \sigma_a y \, h_a \, dy + \int_{-w_b}^{0} \sigma_b y \, h_b \, dy = 0,$$

which can be solved to obtain K and L_*. Finally, we note that since in our physical system

$h_a = h_b$, Eqns. (S40) simplify to

$$\int_0^{w_a} \sigma_a \, dy + \int_{-w_b}^{0} \sigma_b \, dy = 0, \tag{S41}$$
$$\int_0^{w_a} \sigma_a y \, dy + \int_{-w_b}^{0} \sigma_b y \, dy = 0.$$

Linear model. For a linear elastic material, the axial stress in the strips is given by

$$\sigma = \begin{cases} E\left(\dfrac{L_* + y\theta}{L_a} - 1\right), & y > 0 \text{ for strip } a \\ \\ E\left(\dfrac{L_* + y\theta}{L_b} - 1\right), & y < 0 \text{ for strip } b \end{cases} \tag{S42}$$

Substitution of Eqns. (S42) into the equilibrium equations (S41) yields

$$\int_0^{w_a} \left(\frac{L_* + y\theta}{L_a} - 1\right) dy + \int_{-w_b}^{0} \left(\frac{L_* + y\theta}{L_b} - 1\right) dy = 0, \tag{S43}$$
$$\int_0^{w_a} \left(\frac{L_* + y\theta}{L_a} - 1\right) dy + \int_{-w_b}^{0} \left(\frac{L_* + y\theta}{L_b} - 1\right) dy = 0.$$

For the sake of simplicity, here we neglect the effect of the prestrain on the width of the strips and assume $w_a = w_b = w$, so that Eqns. (S43) reduce to

$$L_* \left(\frac{2}{L_a} + \frac{2}{L_b}\right) + \theta \left(\frac{w}{L_a} - \frac{w}{L_b}\right) - 4 = 0, \tag{S44}$$
$$L_* \left(\frac{3}{L_a} - \frac{3}{L_b}\right) + \theta \left(\frac{2w}{L_a} + \frac{2w}{L_b}\right) = 0,$$

from which L_*, θ and K can be obtained as

$$\begin{aligned} L_* &= L_a \frac{8\,(2+\chi)}{2 + 14\chi + (\chi+1)^2}, \\ \theta &= \frac{L_a}{w} \frac{12\chi}{2 + 14\chi + (\chi+1)^2}, \\ K &= \frac{1}{w} \frac{12\chi}{8\,(2+\chi)}. \end{aligned} \tag{S45}$$

where $\chi = L_a/L_b - 1$ denotes the pre-strain.

Non-linear model. Following Huang et al. [1], we assume a fully incompressible elastomer (i.e. $\det(\mathbf{F}) = 1$), so that the state of deformation in each strip is fully characterized by

$$\mathbf{F} = \text{diag}(\lambda, 1/\sqrt{\lambda}, 1/\sqrt{\lambda}), \tag{S46}$$

where $\mathbf{F}$ is the deformation gradient and λ is the stretch

$$\lambda = \epsilon + 1 = \begin{cases} \dfrac{\theta(R+y)}{L_a} = \dfrac{L_* + y\theta}{L_a}, & y > 0 \text{ for strip } a, \\ \\ \dfrac{\theta(R+y)}{L_b} = \dfrac{L_* + y\theta}{L_b}, & y < 0 \text{ for strip } b. \end{cases} \tag{S47}$$

To capture the response of the elastomeric strips, we use the incompressible Gent model [10], so that the uniaxial stress is given by

$$\sigma = \left(\lambda^2 - \frac{1}{\lambda}\right) \frac{\mu J_m}{J_m - I_1 + 3}, \tag{S48}$$

where $\mu = E/3$ is the initial shear modulus, J_m is a constant related to the strain saturation of the material and $I_1 = \lambda^2 + 2/\lambda$. Moreover, the width of each strip entering the evaluation of the integrals in Eqns. (S40) is calculated using the longitudinal stretch at the mid-plane in the current state,

$$w_a = \frac{w}{\sqrt{\lambda_a(y = w_a/2)}}, \quad w_b = \frac{w}{\sqrt{\lambda_b(y = -w_b/2)}}. \tag{S49}$$

Unlike the linear model, Eqns. (S40), (S48) and (S49) cannot be solved analytically, so the trust-region-dogleg algorithm within Matlab software is used to solve numerically for K and L_*.

Results. We consider a bi-strip characterized by $w = 3\,mm$ and $L_a = 500\,mm$. In Fig. S9 we report the evolution of K and L_* as a function of the pre-strain χ for both the linear and non-linear model. The results show a good agreement between the two models. Therefore, for the sake of simplicity the linear model will be used in the stability analysis described in the following sections.

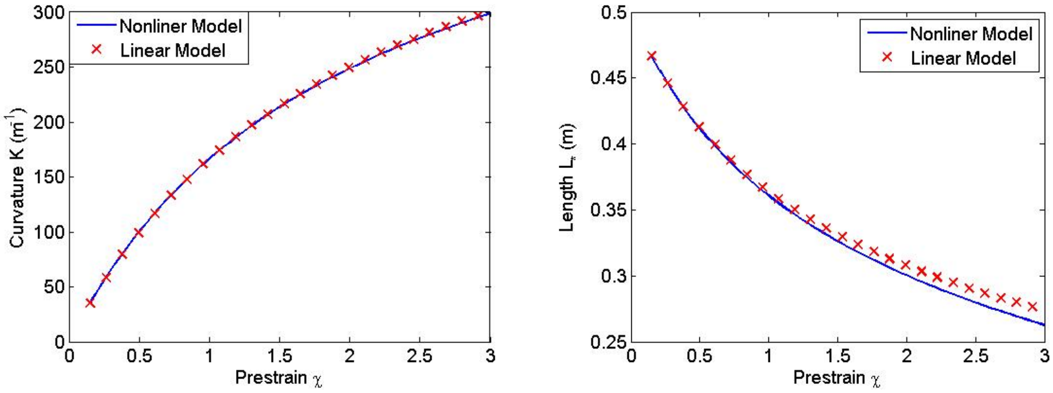

Figure S9: The curvature K and the length L_* as a function of the prestrain ($\chi = L_a/L_b - 1$) for a bi-strip with h=3mm, w=3mm, $L_a = 500mm$. The continuous blue line and the red markers correspond to the predictions from the linear and non-linear model, respectively.

S3.2.2 Equilibrium configurations of the equivalent rod

We now seek solutions of the static equilibrium equations (S24) for the equivalent rod with homogeneous rectangular cross section with edges $2w$ and h, length L_* and intrinsic curvature K given by (S45). Our starting point is the fully stretched state obtained by applying the tensile force P. Here, we first show that both the straight and helical configurations are equilibrium configurations for the system and then demonstrate that for large values of applied tension P the straight configuration is energetically favorable, while below a critical tensile force P_{cr}, the helical configuration grows.

Straight configuration A straight configuration (without twist) is characterized by,

$$\mathbf{d}_1 = \mathbf{e}_1,\ \mathbf{d}_2 = \mathbf{e}_2,\ \mathbf{d}_3 = \mathbf{e}_3, \tag{S50}$$

so that

$$\kappa_1 = \kappa_2 = \kappa_3 = 0, \tag{S51}$$

where $\mathbf{e}_i$ is the basis director of the global coordinate system. Substitution of Eqns. (S50) and (S51) into the static equilibrium equations (S24) yields

$$F_3' = 0,\quad F_1 = F_2 = 0. \tag{S52}$$

Therefore, the rod is subjected to a uniform internal force $\mathbf{F} = P\,\mathbf{d}_3$, where P represents the loading parameter during the releasing process. Moreover, the constitutive relation (S20) implies that

$$\mathbf{M} = -E\,I_1 K \mathbf{d}_1. \tag{S53}$$

Conclusively, a straight rod with internal force $\mathbf{F} = P\,\mathbf{d}_3$ and moment $\mathbf{M} = -E\,I_1 K\mathbf{d}_1$ is an equilibrium state. As a result, the energy density (total energy per length) of the straight configuration is given by

$$\mathcal{E} = \frac{1}{2} E I_1 K^2 - P. \tag{S54}$$

Helical configuration An helical configuration with curvature κ and torsion τ is defined by the position vector

$$\mathbf{x} = \frac{\kappa}{\gamma^2}\sin(\gamma s)\mathbf{e}_1 + \frac{\kappa}{\gamma^2}\left(\cos(\gamma s) - 1\right)\mathbf{e}_2 + \frac{\tau}{\gamma} s\, \mathbf{e}_3, \tag{S55}$$

where $\gamma = \sqrt{\tau^2 + \kappa^2}$. If the helix is untwisted [1], the director basis can be calculated as

$$\begin{aligned}
\mathbf{d}_3 &= \mathbf{x}' = \frac{\kappa}{\gamma}\cos(\gamma s)\mathbf{e}_1 - \frac{\kappa}{\gamma}\sin(\gamma s)\mathbf{e}_2 + \frac{\tau}{\gamma}\mathbf{e}_3, && \text{(S56)}\\
\mathbf{d}_2 &= \frac{\mathbf{d}_3'}{\|\mathbf{d}_3'\|} = -\sin(\gamma s)\mathbf{e}_1 - \cos(\gamma s)\mathbf{e}_2, && \text{(S57)}\\
\mathbf{d}_1 &= \mathbf{d}_2 \times \mathbf{d}_3 = -\frac{\tau}{\gamma}\cos(\gamma s)\mathbf{e}_1 + \frac{\tau}{\gamma}\sin(\gamma s)\mathbf{e}_2 + \frac{\kappa}{\gamma}\mathbf{e}_3, && \text{(S58)}
\end{aligned}$$

so that the curvature $\boldsymbol{\kappa}$ is given by

$$\boldsymbol{\kappa} = \kappa\mathbf{d}_1 + \tau\mathbf{d}_3. \tag{S59}$$

Substitution of Eqns. (S59) into the equilibrium equations (S24) yields

$$\begin{aligned}
F_2 = 0,\quad F_1' = F_3' = 0,\quad -F_3\kappa + F_1\tau = 0, && \text{(S60)}\\
-GJ\tau\kappa + EI_1(\kappa - K)\tau + F_1 = 0. && \text{(S61)}
\end{aligned}$$

Therefore, the force $\mathbf{F}$ in the global coordinates can be written as

$$\mathbf{F} = F_1\mathbf{d}_1 + F_2\mathbf{d}_2 + F_3\mathbf{d}_3 = \frac{F_1\kappa + F_3\tau}{\gamma}\mathbf{e}_3 = P\mathbf{e}_3, \tag{S62}$$

indicating that there are no forces acting in the directions spanned by $\mathbf{e}_1$ and $\mathbf{e}_2$. This is consistent with the experimental setup, where only a tensile force in the longitudinal direction

[1] It has been proved that only untwisted helix can satisfy the equilibrium equations [2].

spanned by $\mathbf{e}_3$ is applied. Moreover, Eqns. (S60)-(S62) indicate that in an equilibrated helix the values of torsion τ, curvature κ and applied force P are related through

$$-GJ\tau\kappa + EI_1(\kappa - K)\tau + \frac{\kappa}{\gamma}P = 0. \tag{S63}$$

Therefore, a helical rod satisfying Eqn. (S63) is also an equilibrium configuration. It is important to note that since in our physical experiments we only control the applied force P, Eqn. (S63) is not sufficient to determine the curvature κ and torsion τ characterizing the helical configuration. To evaluate the evolution of κ and τ as a function of the applied force P, we minimize the total energy (density) of the helix

$$\mathcal{E} = \mathcal{E}_b + \mathcal{E}_\tau + \mathcal{U}_p, \tag{S64}$$

where $\mathcal{E}_b$, $\mathcal{E}_\tau$ and $\mathcal{U}_p$ are the bending energy, twisting energy and force potential, respectively:

$$\mathcal{E}_b = \frac{1}{2}EI_1\left(\kappa - K\right)^2, \tag{S65}$$

$$\mathcal{E}_\tau = \frac{1}{2}GJ\tau^2, \tag{S66}$$

$$\mathcal{U}_p = -\frac{P}{L_*}\frac{\tau\, L_*}{\gamma}, \tag{S67}$$

with $\tau\, L_*/\gamma$ being the end-to-end distance of the helix. The energy minimization criterion requires that

$$\frac{\partial\mathcal{E}}{\partial\kappa} = 0 \quad : \quad EI_1(\kappa - K) + P\frac{\kappa\tau}{\gamma^3} = 0, \tag{S68}$$

$$\frac{\partial\mathcal{E}}{\partial\tau} = 0 \quad : \quad GJ\tau - P\frac{\kappa^2}{\gamma^3} = 0, \tag{S69}$$

which can be solved to obtain κ and τ as a function of the applied force P. Eliminating P from Eqns. (S68) and (S69), we have

$$EI_1(\kappa - K)\kappa + GJ\tau^2 = 0, \tag{S70}$$

from which we can calculate κ as

$$\kappa = \frac{1}{2}\left(K - Q\right), \text{ with } Q = \sqrt{K^2 - \frac{4GJ\tau^2}{EI_1}}. \tag{S71}$$

Substitution of Eqn. (S71) into Eqn. (S68) yields

$$\frac{1}{2}EI_1\left(-K + Q\right) + \frac{P\tau\left(K + Q\right)}{2\left(\tau^2 + \frac{1}{4}\left(K + Q\right)^2\right)^{3/2}} = 0, \tag{S72}$$

which can be solved numerically to obtain the curvature κ as a function of P.

Note that the constitutive equation (S20) indicates that a stable helix is subjected to a twisting moment $GJ\tau$ and a bending moment $EI_1(\kappa - K)$, which are not controlled in our experiments. In fact, since experimentally both ends are free to rotate, the twisting moment cannot be supported by our samples [2]. Furthermore, we note that in a perfect helix the ends of

[2] Note that the bending moment is approximately supported by the non-uniform stress distribution in the bi-strip system.

the rod are not aligned. Such misalignment cannot be supported by our physical samples during the releasing process, since the gravity acts against it (see the experimental section for more details). Therefore, during the growth of the helix a transition region dominated by boundary effects near the strip ends may form and non-negligible dynamic effects may arise. Nonetheless, our experiments show that the transitional region near the ends is very short. Moreover, since the releasing process is slow, no significant dynamics effects are observed, so that Eqns. (S71)-(S72) can be used to describe the response of our system with good accuracy.

Transition from straight to helical configurations Although both straight and helical configurations represent equilibrium states for the equivalent rod, our experimental observations suggest that for large values of the applied force P the straight configuration is stable. However, when the applied force P is gradually released, the rod (a thick one) is found to evolve from a straight to a helical configuration.

Interestingly, we note that for large values of the applied force P, Eqn. (S72) does not admit a real, positive solution κ. Therefore, for large values of P helical configurations are not supported by the rod. However, a critical value of the applied force P_{cr} can be identified for which Eqn. (S72) starts to admit a real and positive solution κ. Hence, for $P < P_{cr}$ helical configurations are expected to emerge. The critical force P_{cr} can be obtained by taking the limit $\kappa \to 0$ in Eqn. (S72),

$$P_{cr} = \frac{(EI_1K)^2}{GJ}. \tag{S73}$$

Finally, to further highlight the transition from straight to helical configurations, we compare the energies of both states as a function of the applied force P. The results reported in Fig. S10 clearly show that when $P < P_{cr}$ the helix has lower energy than the straight configuration.

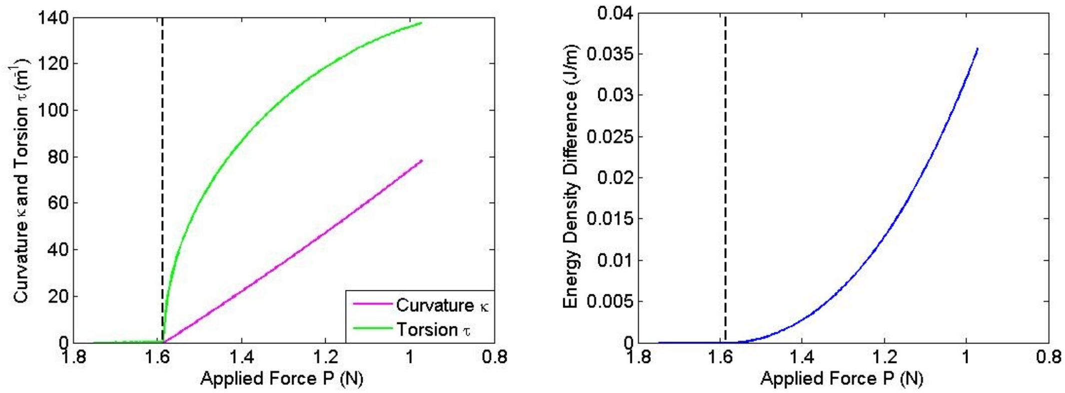

Figure S10: Left: Curvature κ and torsion τ as a function of the applied force P for a bi-strip with $\chi = 1.5$, $h = 6$mm, $w = 3$mm, $L_a = 500$mm. Note that these parameters result in a rod with $K = 0.2143\text{mm}^{-1}$ and $L_* = 331.4$mm. Right: Energy density difference $\mathcal{E}_{straight} - \mathcal{E}_{helix}$ as a function of the applied force P for the straight and helical configurations. In both plots the vertical dashed line correspond to P_{cr}.

S3.2.3 Stability of the equivalent rod

In the section above, we have proved that as the applied force P is gradually released at a critical point the straight configuration becomes unstable and the rod assumes an helical shape.

However, in the experiments during the release process we observe the formation not only of helices, but also of hemihelices with multiple reversal of chirality. Here, we show that all these complex 3D shapes can be captured conducting the general stability analysis described in Section S3.1.4. Interestingly, the analysis reveals that the helical transition studied in Section S3.2.2 can be recovered as a special case of the bifurcation branches obtained by this general stability analysis.

To understand and analyze the complex shapes observed during the release process in the pre-strained bi-strip, we start by considering a straight rod under tension, so that $\mathbf{F} = P\,\mathbf{d}_3$. The rod has an homogeneous rectangular cross section with edges $2w$ and h and is characterized by a length L_* and intrinsic curvature K given by (S45). In Section S3.2.2 we have shown that the straight configuration characterized by

$$(\kappa_1^{(0)}, \kappa_2^{(0)}, \kappa_3^{(0)}) = (0,0,0), \;\; (F_1^{(0)}, F_2^{(0)}, F_3^{(0)}) = (0,0,P), \;\; (\kappa_1^{(u)}, \kappa_2^{(u)}, \kappa_3^{(u)}) = (K,0,0), \tag{S74}$$

identically satisfies the equilibrium equations (S24). However, the experiments show that a critical tension exists for which the straight filament loses its stability and bifurcate into new solutions. To capture the bifurcation, we focus on the first order equilibrium equations (S36) and express the solution $\boldsymbol{\mu}^{(1)}$ as

$$\boldsymbol{\mu}^{(1)} = \mathbf{c}\exp(\mathrm{i}\omega_n s), \tag{S75}$$

where $\mathbf{c}$ is the amplitude vector and $\omega_n = n\,\pi/L_*$ is the angular frequency of the corresponding mode. Substituting Eqns. (S74) and (S86) into (S36), the first order equilibrium equations can be rewritten as

$$\mathbf{L}\,\mathbf{c} = \mathbf{0}, \tag{S76}$$

where $\mathbf{L}$ is given by

$$\mathbf{L} = \begin{pmatrix} 0 & -P\omega_n^2 & 0 & -\omega_n^2 & 0 & 0 \\ P\omega_n^2 & 0 & 0 & 0 & -\omega_n^2 & 0 \\ 0 & 0 & 0 & 0 & 0 & -\omega_n^2 \\ -I_1E\omega_n^2 & 0 & 0 & 0 & -1 & 0 \\ 0 & -I_2E\omega_n^2 & -\mathrm{i}EI_1K\omega_n & 1 & 0 & 0 \\ 0 & \mathrm{i}EI_1K\omega_n & -GJ\omega_n^2 & 0 & 0 & 0 \end{pmatrix}. \tag{S77}$$

Note that $\mathbf{L}$ not only depends on the material properties, but also on the applied force P. In general, for large values of P the matrix $\mathbf{L}$ is non-singular, so that only the trivial straight solution (i.e. $\mathbf{c}$=$\mathbf{0}$) exists. However, during the release process non-trivial solutions for $\boldsymbol{\mu}^{(1)}$ are supported when

$$\det(\mathbf{L}) = -\omega_n^2\left[\omega_n^2\left(P + (E\omega_n^2)I_1\right)\right]\left[GJ\omega_n^4\left(P + E\omega_n^2 I_2\right) - E^2\omega_n^4 I_1^2 K^2\right] = 0. \tag{S78}$$

Therefore, the critical value of the applied force P_{cr} is obtained as

$$P_{cr} = \frac{(EI_1K)^2}{GJ} - EI_2\omega_n^2 = \frac{(EI_1K)^2}{GJ} - EI_2\frac{n^2\pi^2}{L_*^2}. \tag{S79}$$

Next, we construct the solution corresponding to different modes. First, we use Eqn. (S87) to calculate the eigenvector $\mathbf{c}$ corresponding to a given pair (P_{cr}, ω_n). Then, we substitute the solution Eqn. (S75) in Eqn. (S38) to determine the mode shape up to the first order. Moreover,

to obtain the mode shape more accurately, we solve the second order equilibrium equations (S37), where

$$\mathbf{H}_2 = \exp(2\mathrm{i}\omega_n s)\cdot \begin{pmatrix} -2(Pc_1c_3 - 2c_3c_5 + 2c_2c_6)\omega_n^2 \\ -2(Pc_2c_3 + 2c_3c_4 - 2c_1c_6)\omega_n^2 \\ 2\left[P(c_1^2+c_2^2) + 2c_2c_4 - 2c_1c_5\right]\omega_n^2 \\ \mathrm{i}EI_1Kc_2^2\omega_n + \mathrm{i}EI_1Kc_3^2\omega_n - c_3[c_4 + 2c_2(-EI_2+GJ)\omega_n^2] \\ -\mathrm{i}EI_1Kc_1c_2\omega_n - c_3\left[c_5 - 2c_1(-EI_1+GJ)\omega_n^2\right] \\ c_2c_5 + c_1\left[c_4 - 2I_2c_2E\omega_n^2 + I_1(-\mathrm{i}EKc_3\omega_n + 2Ec_2\omega_n^2)\right] \end{pmatrix}, \tag{S80}$$

c_i being the i-th component of the vector $\mathbf{c}$ determined from the first order equilibrium. The solution $\boldsymbol{\mu}^{(2)}$ can be obtained as

$$\boldsymbol{\mu}^{(2)} = \mathbf{L}^{-1}\cdot\mathbf{H}_2, \tag{S81}$$

and used to determine the director basis $\mathbf{d}_i$ up to the second order. Finally, the position vector for each bifurcation mode is obtained using Eqn. (S38) as

$$\mathbf{x} = \begin{pmatrix} -\dfrac{GJX_n\sin(\omega_n s)}{EI_1K} \\ \\ \dfrac{G^2J^2(2EI_1+EI_2-GJ)X_n^2\omega_n^2(\cos(2\omega_n s)-1)}{2E^3I_1^3K^3 + 2E^2GI_1(4I_1-I_2)JK\omega_n^2} \\ \\ s - \dfrac{G^2J^2X_n^2\omega_n\sin(2\omega_n s)}{4E^2I_1^2K^2} \end{pmatrix}, \tag{S82}$$

where X_n is the mode amplitude. In Fig. S11 we report the shapes of modes characterized by $n = 1,\ 4,\ 7$. The modes clearly resemble the 3D curls (hemi-helices) observed in the experiments and consist of multiple, periodic and alternating helical sections of opposite chiralities, separated by perversions. The first mode is characterized by two regions of opposite chiralities with one perversion, the second mode consists of a sequence of three regions of opposite chiralities and two perversions, the third mode consists of four regions of opposite chirality with three perversions and so on with the number of perversions monotonically increasing linearly with the mode number.

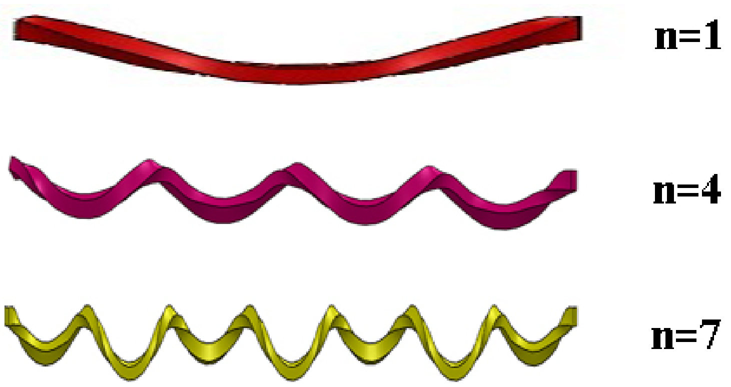

Figure S11: Reconstructions of modes characterized by $n = 1,\ 4,\ 7$.

The special case of the helix Experimental observations clearly show that for thick strips, helices and not hemihelices form during release. Here, we show that at the bifurcation onset the helix can be recovered as a special case of the hemi-helix.

In fact, at the onset of bifurcation the magnitude of both the curvature and the torsion of the helix is small, so that $\kappa \ll 1$ and $\tau \ll 1$. Substituting Eqn. (S71) into Eqns. (S55), expanding the nonlinear terms in Taylor series and retaining only first order terms, the position vector of the helix can be written as

$$\begin{aligned} x_1 &= \frac{\kappa}{\gamma^2}\sin(\gamma s) \sim \frac{GJ}{EI_1 K}\tau s + O(\tau^3) \\ x_2 &= \frac{\kappa}{\gamma^2}(\cos(\gamma s) - 1) \sim O(\tau^2) \\ x_3 &= \frac{\tau}{\gamma} s \sim s + O(\tau^2) \end{aligned} \quad \text{(S83)}$$

On the other hand, if we assume that $\omega_n = n\pi/L_* \ll 1$ in Eqns. (S82), expand the nonlinear terms in Taylor series and retain only first order terms, the position vector of the hemihelix can be expressed as

$$\begin{aligned} x_1 &= -\frac{GJX_n \sin(\omega_n s)}{EI_1 K} \sim -\frac{GJ}{EI_1 K} X_n \omega_n s + O(\omega_n^3) \\ x_2 &= \frac{G^2 J^2 (2EI_1 + EI_2 - GJ) X_n^2 \omega_n^2 (\cos(2\omega_n s) - 1)}{2E^3 I_1^3 K^3 + 2E^2 G I_1 (4I_1 - I_2) JK\omega_n^2} \sim O(\omega_n^2) \\ x_3 &= s - \frac{G^2 J^2 X_n^2 \omega_n \sin(2\omega_n s)}{4E^2 I_1^2 K^2} \sim s + O(\omega_n^2) \end{aligned} \quad \text{(S84)}$$

It is easy to see that Eqns. (S83) and (S84) coincide if $\tau = -X_n \omega_n$. Therefore, our analysis reveals that at the onset of bifurcation the helix can be described as an hemihelix characterized by small mode number n.

This can be further illustrated by directly comparing the mode shapes obtained using Eqns. (S82) for different values of n, as shown in Fig. S12. Since an hemi-helix consists of helical sections of opposite chiralities separated by perversions, if $n < 1$ the perversion lies outside the strip and the rod deforms into a single helical segment. Therefore, we expect to observe the formation of helices during the release process if $n < 1$.

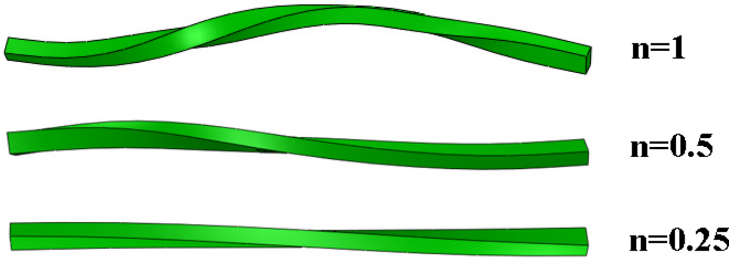

Figure S12: Reconstructions of modes characterized by n = 1, 0.5, 0.25. Note that for case $n = 1$, the reconstruction shows the segment between the perversions, so that the perversion lies at each end of the strip.

Finally, by taking the limit for $\omega_n \to 0$ in Eqn. (S79) we obtain

$$P_{cr} = \frac{(EI_1 K)^2}{GJ}, \quad \text{(S85)}$$

which coincides with the critical force previously calculated for an helix (see Eqn. (S73)). This further confirms the close relationship between hemi-helices and helices.

S3.2.4 Mode selection

The stability analysis conducted in the previous section shows when $P \leq P_{cr}$ the straight configuration is unstable and the rod assumes a 3D shape. In Fig. S13 we report the evolution of P_{cr} as a function of the dimensionless cross-sectional parameter h/w for different modes (i.e. different values of n).

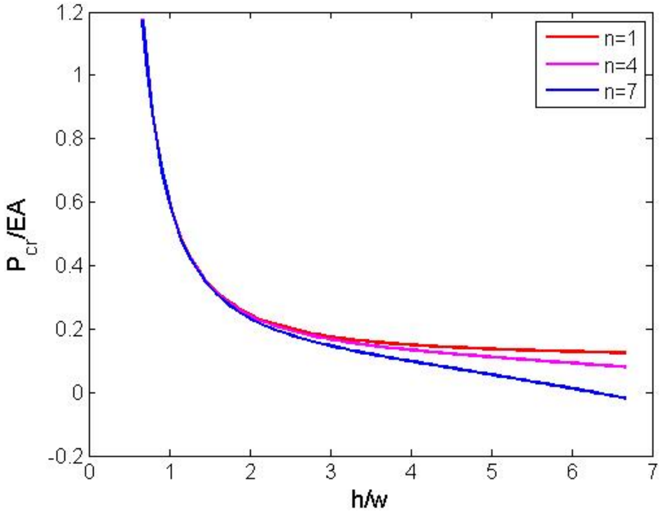

Figure S13: Critical load P_{cr} as a function of the cross-sectional aspect ratio h/w for different mode numbers n. A bi-strip characterize by $w = 3$mm, $\chi = 1.5$ and $L = 500$mm is considered.

The results clearly show that the mode characterized by $n = 1$ is always the first to be excited. However, it is important to note that for low values of h/w the modes are closely spaced, while as h/w increases, the critical values for different modes become more and more separated. Therefore, for a thick strip low number modes are more likely to dominate, since they may evolve before higher number modes are triggered. Differently, for low values of h/w we do not expect to necessarily observe the mode with $n = 1$, but that which grows faster than the others.

Here, to determine the mode selected by the rod with a simple analysis, we assume that the fastest growing mode at the onset of the instability is the one that dominates. Although this approach neglects the contribution of geometric non-linearities and the possible interactions between different modes, it has already been successfully used to determine the mode selected by rods in a variety of contexts [5, 15, 16, 17, 18]. Moreover, we will show that the predictions obtained using this simple analysis nicely agree with both our experimental and numerical results.

Therefore, to determine the mode selected by the rod we focus on the first order equilibrium equations (S36) and express the solution $\boldsymbol{\mu}^{(1)}$ as

$$\boldsymbol{\mu}^{(1)} = \mathbf{c} \exp(\sigma t + \mathrm{i}\omega_n s), \tag{S86}$$

where σ is the growth rate of the bifurcation mode. Substituting Eqns. (S74) and (S86) into

(S36), the first order equilibrium equations can be rewritten as

$$\mathbf{L}\,\mathbf{c} = \mathbf{0}, \tag{S87}$$

where $\mathbf{L}$ is given by

$$\mathbf{L} = \begin{pmatrix} 0 & -A\rho\sigma^2 - P\omega_n^2 & 0 & -\omega_n^2 & 0 & 0 \\ A\rho\sigma^2 + P\omega_n^2 & 0 & 0 & 0 & -\omega_n^2 & 0 \\ 0 & 0 & 0 & 0 & 0 & -\omega_n^2 \\ -I_1(\rho\sigma^2 + E\omega_n^2) & 0 & 0 & 0 & -1 & 0 \\ 0 & -I_2(\rho\sigma^2 + E\omega_n^2) & -\mathrm{i}EI_1K\omega_n & 1 & 0 & 0 \\ 0 & \mathrm{i}EI_1K\omega_n & -(I_1+I_2)\rho\sigma^2 - GJ\omega_n^2 & 0 & 0 & 0 \end{pmatrix}. \tag{S88}$$

During the release process non-trivial solutions for $\boldsymbol{\mu}^{(1)}$ are supported when

$$\begin{aligned}\Delta(\sigma,\,\omega_n) \;=\; & \det(\mathbf{L}) = -\omega_n^2\left(\rho\sigma^2 A + \omega_n^2\left(P + (\rho\sigma^2 + E\omega_n^2)I_1\right)\right)\cdot \\ & \left(\left(\rho\sigma^2 I_1 + \rho\sigma^2 I_2 + GJ\omega_n^2\right)\left(\rho\sigma^2 A + \omega_n^2\left(P + (\rho\sigma^2 + E\omega_n^2)I_2\right)\right) - E^2\omega_n^4 I_1^2 K^2\right) = 0.\end{aligned} \tag{S89}$$

Therefore, to determine the mode selected by the rod we calculate the growth rate σ by solving equation (S89) for a given value of P and ω_n. When $P < P_{cr}$ solutions with positive real values of σ are found, identifying perturbations that grow exponentially with time. These modes are the solutions that grow exponentially from small perturbations and are those observed in experiments. Intuitively, as a consequence of their exponential growth, we expect the modes with the highest growth rate σ to dominate the morphological evolution. In contrast, for $P > P_{cr}$ solutions with imaginary or negative σ are obtained; these will be of the order of the perturbation itself, cannot grow and hence will not be observed. Finally, when $P = P_{cr}$ we find that $\sigma = 0$ and the solution reduces to that considered in the stability analysis in Section S3.2.3.

In Fig. S14 we report the growth rate as a function of the mode number n for strips with different aspect ratio h/w. The results clearly show that the fastest growing mode in a thick strip with $h/w = 2$ is characterized by $n = 3$. Differently, for a thin strip with $h/w = 1$ the mode with $n = 9$ is the fastest to evolve and is expected to dominate. These observations nicely agree with the experimental results reported in Fig. 3 in the main text, where it is clearly shown that the mode number monotonically decreases as a function of h/w.

S3.2.5 Results and discussion

The analyses conducted in the previous sections show that during the release process a critical value of applied force P_{cr} exists below which the straight configuration is unstable. For $P < P_{cr}$ the analysis predicts the formation of hemi-helices consisting of helical sections of opposite chiralities separated by perversions. To determine the number n of perversions that form in the rod, the growth rate σ for different modes can be calculated and compared. The mode characterized by the highest growth rate σ is expected to grow faster and to dominate.

We perform a series of stability analyses on rods characterized by different values of prestrain χ and cross-sectional aspect ratio h/w and find the mode n that has the maximum growth rate σ. The results are reported in Fig. S15 as contour map. The color in the contour plot represents the associated value of n for which the growth rate is maximum and therefore correspond to the number of perversions n_p we expect to observe in the system. This parametric study reveals that the number of perversions in the rod after bifurcation is only moderately affected by the pre-strain χ, while the aspect ratio h/w is found to have a significant effect. In thin strips with $h/w \simeq 1$ the formation of 10 perversions is observed. The number of perversions n_p is then

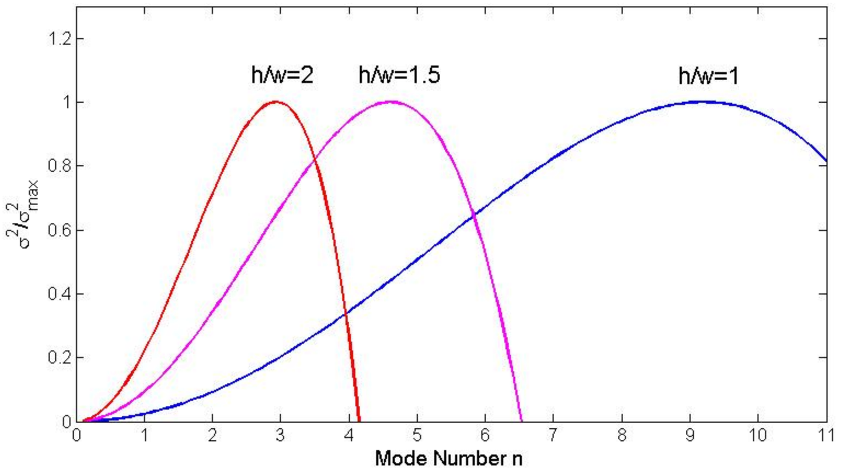

Figure S14: Growth rate σ as a function of the mode number n for three different strips characterized by $h/w = 1$, 1.5, 2, $w = 3$mm , $\chi = 1.5$ and $L = 500$mm. The growth rate is determined for an applied force $P = 0.981 P_{cr}$.

found to monotonically decrease as a function of h/w. In particular, the dashed red line in the plot marks the configurations for which $n_p = 1$. As highlighted in the previous section, if $n_p < 1$ the perversion lies outside the rod, so that the system deforms into a single helical segment and the formation of helices is expected during the release process. Therefore, the red line defines the boundary between hemi-helices and helices. It is worth noting that, since the growth rate σ depends also on the applied force P, the number of perversions predicted by the analysis is also a function of P. To clarify this aspect in Fig. S16 we report the contour map for n_p calculated for different values of P. Although the value of P is found to slightly alter n_p and therefore to shift the phase boundary between hemihelices and helices, all the contour maps share the same key features.

In conclusion, the results of our analysis not only enable us to explain the evolution of the number of perversions as a function of χ and h/w observed experimentally in the bi-strip, but also, and most importantly, capture the transition from hemihelices to helices.

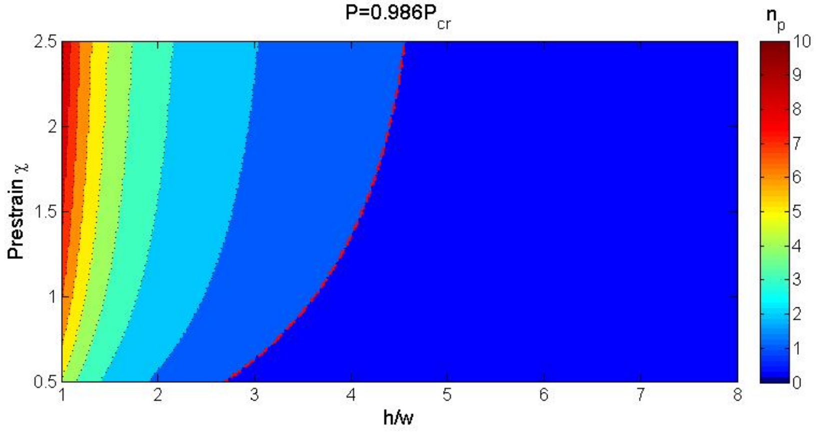

Figure S15: Contour plots showing the value of n for which the growth rate is maximum as function to χ and h/w. The growth rates are calculated for $P = 0.986P_{cr}$. Black dotted lines show the boundaries between modes with different number of perversions n_p, while the red dashed line corresponds to $n_p = 1$ and separates hemi-helices (to its left) from helices (to its right).

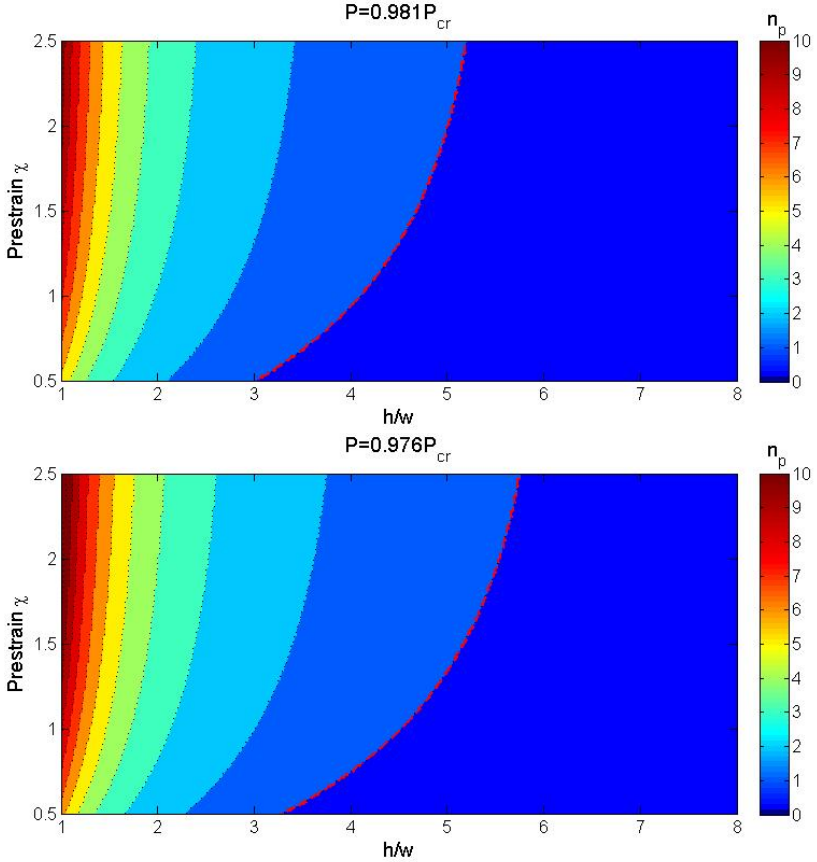

Figure S16: Contour plots showing the values of n_p for different values of applied force, $P = 0.981P_{cr}$ and $P = 0.976P_{cr}$. P is found to slightly alter the boundaries between modes characterized by different values of n_p and therefore to shift the phase boundary between hemihelices and helices. However, all the contour maps show the same key features.

References

[1] Huang J, Liu J, Kroll B, Bertoldi K, Clarke DR (2012) Spontaneous and deterministic three-dimensional curling of pre-strained elastomeric bi-strips. *Soft Matter* 8:6291-6300.

[2] McMillen T, Goriely A (2002) Tendril perversion in intrinsically curved rods. *J. Nonlinear Sci.* 12:241-281.

[3] Coleman BD, Dill EH, Lembo M, Lu Z, Tobias I (1993) On the dynamics of rods in the theory of Kirchhoff and Clebsch. *Arch Rational Mech Anal* 121:339-359.

[4] Young W, Budynas R (2002) Roark's formulas for sress & strain, 7th Edition.

[5] Goriely A, Tabor M (1997) Nonlinear dynamics of filaments I. Dynamical instabilities. *Physica D* 105: 20-44.

[6] Goriely A, Tabor M (1997) Nonlinear dynamics of filaments II. Nonlinear analysis. *Physica D* 105: 45-61.

[7] Audoly B, Pomeau Y (2010) Elasticity and geometry. *Oxford University Press.*

[8] Bower AF (2009) Applied mechanics of solids. *CRC Press. Taylor and Francis Group.*

[9] Goriely A, Nizette M, Tabor M (2001) On the dynamics of elastic strips. *J Nonlinear Sci* 11:3-45.

[10] Gent AN (1996) A new constitutive relation for rubber. *Rubber Chem Technol* 69:59-61.

[11] Kroner E (1960) Allgemeine Kontinuumstheorie der Versetzungen und Eigenspannungen. *Arch Ration Mech Anal* 4:273-334.

[12] Lee EH (1969) Elastic-Plastic deformation at finite strains. *J Appl Mech* 36:1-6.

[13] Kirchhoff G (1859) Uber das Gleichgewicht und die Bewegung eines unendlich dfinnen elastischen Stabes. *J f reine, angew Math* 56:285-313.

[14] Kirchhoff G (1876) Vorlesungen fiber mathematische Physik. *Mechanik* 28.

[15] Goriely A (2000) The nonlinear dynamics of filaments. *Nonlinear Dynam* 21:101-133.

[16] Patricio P, Adda-Bedia M, Amar MB (1998) An elastica problem: instability of an elastic arch. *Physica D* 124:285-295.

[17] Zhou H, Ou-Yang Z (1999) Spontaneous curvature-induced dynamical instability of Kirchhoff filaments: Application to DNA kink deformations. *J Chem Phys* 110:1247-1251.

[18] Valverde J, Van der Heijden GHM (2010) Magnetically-induced buckling of a whirling conducting rod with applications to electrodynamic space tethers. *J Nonlinear Sci* 20:309-339.
